# Rhodium-Catalyzed Oxidative Alkenylation of Anisole: Control of Regioselectivity

**DOI:** 10.1021/acs.organomet.4c00155

**Published:** 2024-06-13

**Authors:** Christopher W. Reid, T. Brent Gunnoe

**Affiliations:** Department of Chemistry, University of Virginia, Charlottesville, Virginia 22904, United States

## Abstract

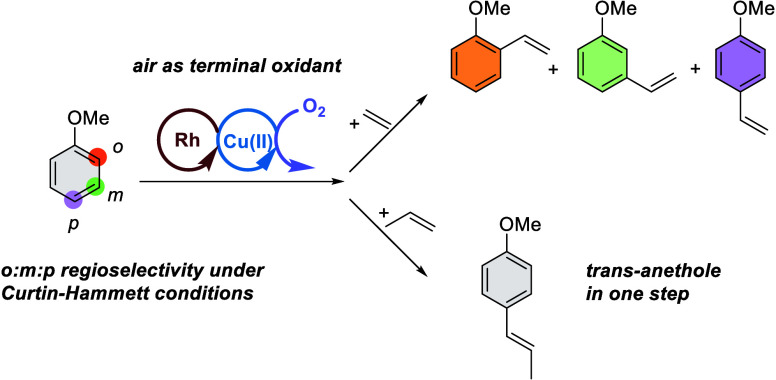

We report the conversion of anisoles and olefins to alkenyl anisoles via a transition-metal-catalyzed arene C–H activation and olefin insertion mechanism. The catalyst precursor, [(η^2^-C_2_H_4_)_2_Rh(μ-OAc)]_2_, and the in situ oxidant Cu(OPiv)_2_ (OPiv = pivalate) convert anisoles and olefins (ethylene or propylene) to alkenyl anisoles. When ethylene is used as the olefin, the *o*/*m*/*p* ratio varies between approximately 1:3:1 (selective for 3-methoxystyrene) and 1:5:10 (selective for 4-methoxystyrene). When propylene is the olefin, the *o*/*m*/*p* regioselectivity varies between approximately 1:8:20 and 1:8.5:5. The *o*/*m*/*p* ratios depend on the concentration of pivalic acid and olefin. For example, when using ethylene, at relatively high pivalic acid concentrations and low ethylene concentrations, the *o*/*m*/*p* regioselectivity is 1:3:1. Conversely, again for use of ethylene, at relatively low pivalic acid concentrations and high ethylene concentrations, the *o*/*m*/*p* regioselectivity is 1:5:10. Mechanistic studies of the conversion of anisoles and olefins to alkenyl anisoles provide evidence that the regioselectivity is likely under Curtin–Hammett conditions.

## Introduction

New methods to synthesize alkenyl anisoles, such as *trans*-anethole, are of importance due to their relevance in medicinal chemistry and the cosmetic industry.^1^ For example, *trans*-anethole has been studied for its antimetastatic,^2−4^ antiedematogenic,^5^ and antioxidant^6^ properties. It can be isolated from natural sources such as fennel oil, anise oils, star anise oil, clove oil, and aniseed;^7−9^ however, isolation from these oils does not always satisfy the demand. Thus, synthetic processes starting from anisole have been developed for the preparation of *trans*-anethole.^1^

**Scheme 1:**
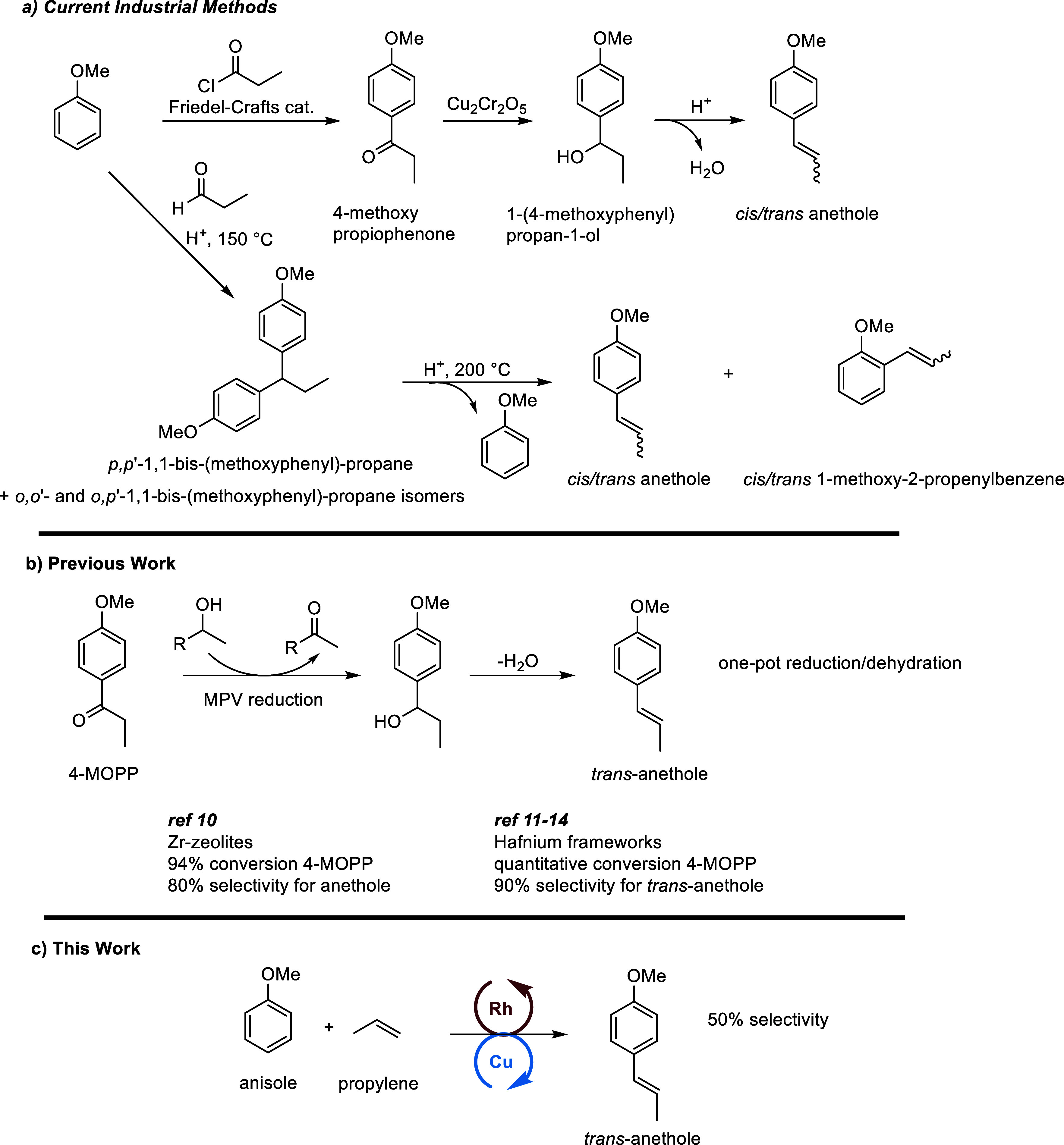
Synthesis of Anethole (a) Current industrial methods for the preparation of anethole. (b) Previous work on the cascade reduction/dehydration of 4-MOPP to *cis*/*trans* anethole. (c) Reported method for the preparation of *trans*-anethole and other alkenyl anisoles by Rh-catalyzed oxidative arene alkenylation.

One industrial method to produce *trans*-anethole utilizes a 3-step process involving Friedel–Crafts acylation of anisole with propionyl chloride to form 4-methoxypropiophenone (4-MOPP), reduction of the ketone to the corresponding alcohol using a copper chromite catalyst, and dehydration under acidic conditions to give a mixture of *cis*- and *trans*-anethole (Scheme 1a).^1^ Alternatively, mixing anisole and propionaldehyde under acidic conditions at 150 °C forms an isomeric mixture of *p,p*′-, *p,o*′-, and *o,o*′-1,1-bis(methoxyphenyl)propanes, which can then be cleaved to yield anisole and a mixture of *cis*- and *trans*-anethole.^1^ The cleavage of the isomeric mixture gives an approximate 66% yield of propenylanisoles based on bis(methoxyphenyl)propanes with an approximate ∼38% selectivity for the *trans*-anethole product. Recently, the conversion of 4-MOPP to *cis*- and *trans*-anethole via a cascade Meerwein–Ponndorf–Verley (MPV) reduction/dehydration process was reported using Zr-containing zeolites achieving 94% conversion of 4-MOPP in 4 h with 80% selectivity for *trans*-anethole.^10^ Additionally, bifunctional organophosphate-hafnium frameworks have been shown to convert 4-MOPP to *trans*-anethole with quantitative conversion of 4-MOPP and 90% selectivity for *trans*-anethole through an MPV reduction/dehydration process.^11−14^

**Scheme 2:**
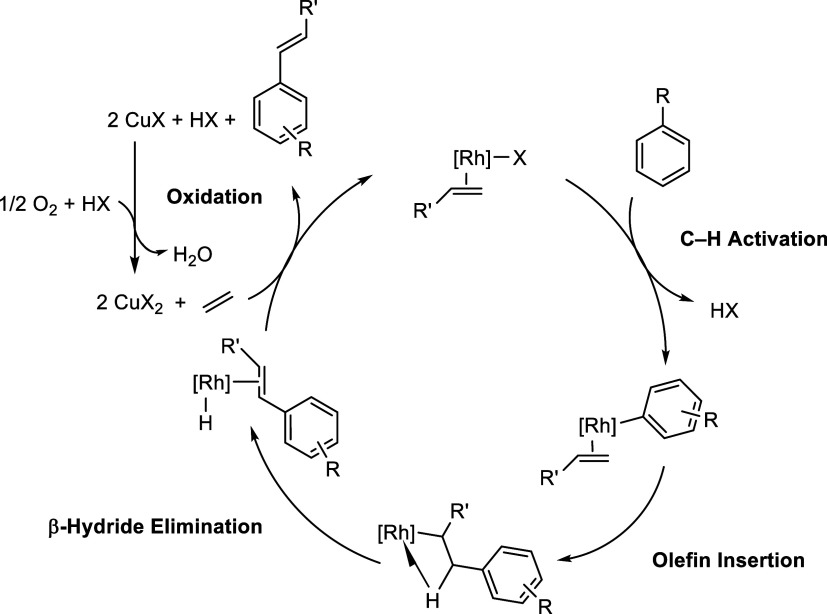
Proposed Mechanism for Transition Metal-Catalyzed Oxidative Arene Alkenylation Using Monosubstituted Arenes and Olefins Using a Rh(I) Catalyst Precursor and a CuX_2_ Oxidant (X = Acetate, Pivalate, and 2-Ethylhexanoate)

Arene alkylation catalysis using hydrocarbons to produce saturated alkyl arenes has previously been reported using Ru,^15−23^ Ir,^24,25^ Pt,^26−32^ and Ni.^33,34^ Oxidative arene alkenylation using hydrocarbons to produce unsaturated alkenyl arenes has been reported using Ru,^35^ Ir,^36^ Pd,^37−42^ and Rh.^41−54^ Previously, we have studied Rh-based arene alkenylation catalysts that operate via C–H activation of the arene and subsequent olefin insertion into the Rh–aryl bond, followed by β-hydride elimination to produce the alkenyl arene.^51^ The use of Cu(II) carboxylates as in situ oxidants allows overall aerobic oxidation since the reduced Cu(I) can be oxidized using dioxygen or unpurified air to regenerate Cu(II) (Scheme 2).^15,41,44−46,48,50,53,54^

Based on our previous studies of oxidative arene alkenylation using [(η^2^-C_2_H_4_)_2_Rh(μ-OAc)]_2_ as a catalyst precursor, we envisioned a process for which anisole and propylene in the presence of the same Rh catalyst precursor and CuX_2_ (X = acetate, pivalate, and 2-ethylhexanoate) oxidant would yield propenylanisoles. Although we have previously reported the alkenylation of substituted arenes (e.g., toluene, trifluorotoluene, and anisole) using [(η^2^-C_2_H_4_)_2_Rh(μ-OAc)]_2_ and Pd(OAc)_2_ precursors and CuX_2_ as an in situ oxidant, we have not completed a study specifically using anisole and ethylene or propylene.^41,54^ Herein, we report the production of alkenyl anisoles from anisole and ethylene or propylene using [(η^2^-C_2_H_4_)_2_Rh(μ-OAc)]_2_ as a catalyst precursor and Cu(OPiv)_2_ (OPiv = pivalate) oxidant. A key focus of our studies was on *o*/*m*/*p* selectivity as a function of reaction conditions.

## Results and Discussion

**Scheme 3:**
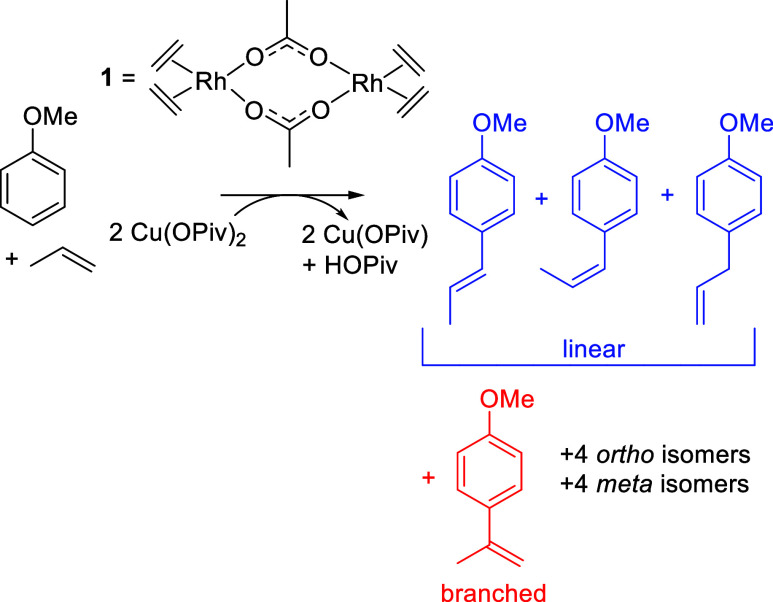
Potential Products of Anisole Alkenylation Using Propylene as the Olefin Catalyzed by Rh Using Cu(OPiv)_2_ as an In Situ Oxidant

With the goal of synthesizing propenylanisoles from anisole and propylene, we sought to optimize reaction conditions (temperature, olefin concentration, CuX_2_ identity, and carboxylic acid concentration). For the conversion of anisole and propylene, there are 12 possible products (Scheme 3). Because of the complications of analyzing 12 isomers of propenylanisoles, we started by optimizing our reaction conditions using ethylene.

**Figure 1:**
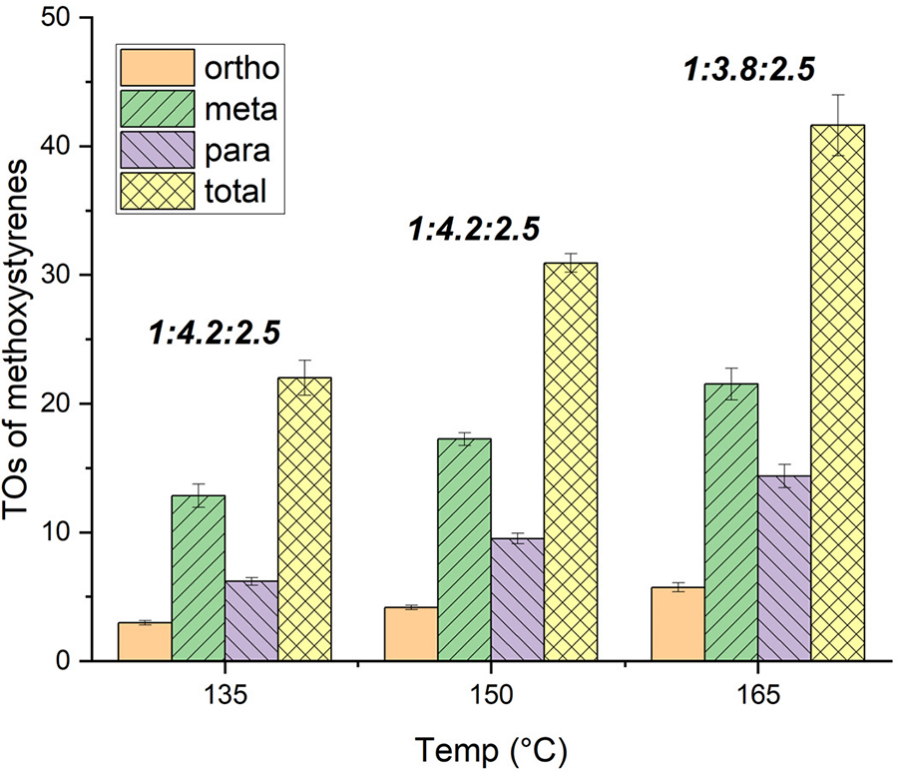
Temperature optimization for oxidative anisole alkenylation catalyzed by [(η^2^-C_2_H_4_)_2_Rh(μ-OAc)]_2_. Reaction conditions: 0.01 mol % of [(η^2^-C_2_H_4_)_2_Rh(μ-OAc)]_2_, 7.5 mL of anisole, 50 psig of ethylene, 120 equiv of Cu(OPiv)_2_, 240 equiv of HOPiv, 4 h, and *x* °C. Catalyst loading is relative to anisole per single Rh atom. Cu(OPiv)_2_ and HOPiv loading relative to a single Rh atom. Bold ratios are *o*/*m*/*p* ratios. HMB used as the internal standard. Error bars represent the standard deviation for a minimum of three independent reactions.

Heating a solution of neat anisole (7.5 mL) with 0.01 mol % of **1** (relative to anisole) in the presence of 120 equiv of Cu(OPiv)_2_ (120 equiv relative to a single Rh atom), 240 equiv of pivalic acid (240 equiv relative to a single Rh atom), and 50 psig of ethylene at 150 °C affords 2-, 3-, and 4-methoxystyrenes in an approximate 1:4.5:2.5 (*o*/*m*/*p*) ratio with 31(1) total turnovers (TOs) after 4 h (Figure 1). The addition of pivalic acid improves the solubility of Cu(OPiv)_2_ in neat anisole. We sought to examine the effect of the temperature on the *o*/*m*/*p* selectivity and TOs (Figure 1). Lowering the temperature to 135 °C affords 22(1) TOs of methoxystyrenes with an *o*/*m*/*p* ratio of 1:4.2:2, which is a slight increase in the selectivity for the 2- and 3-methoxystyrene products compared with reaction at 150 °C. Upon increasing the temperature to 165 °C, we observe 42(2) TOs of methoxystyrenes and a slight decrease in the selectivity for 2- and 3-methoxystyrenes compared to the selectivities for reactions performed at 150 °C with an *o*/*m*/*p* ratio of 1:3.8:2.5.

**Figure 2:**
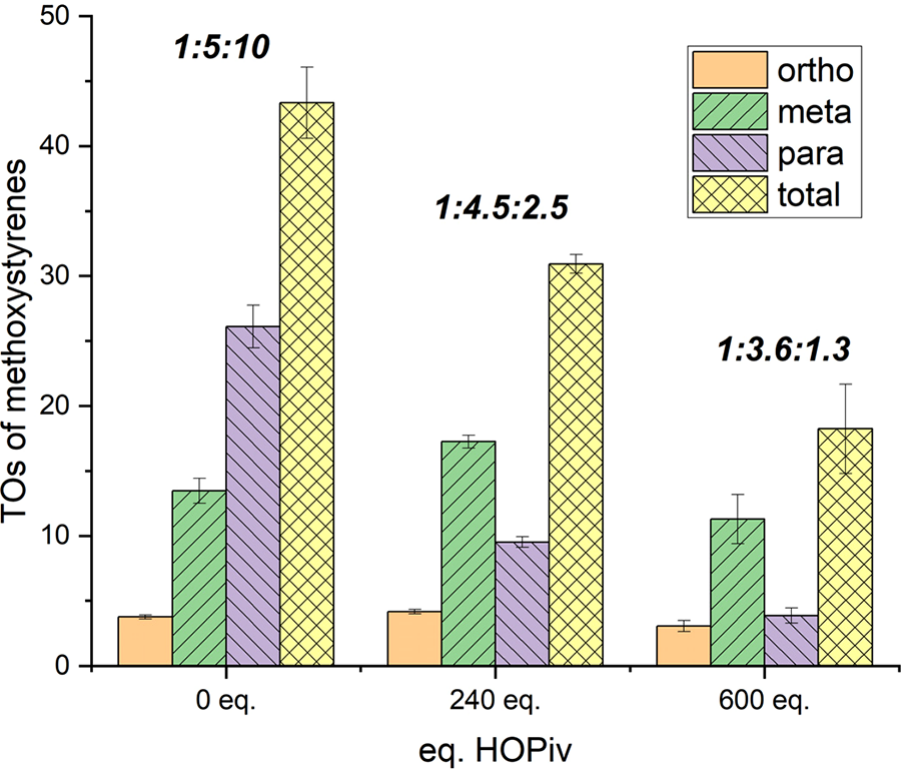
Pivalic acid equivalence optimization for oxidative anisole alkenylation catalyzed by [(η^2^-C_2_H_4_)_2_Rh(μ-OAc)]_2_. Reaction conditions: 0.01 mol % of [(η^2^-C_2_H_4_)_2_Rh(μ-OAc)]_2_, 7.5 mL of anisole, 50 psig of ethylene, 120 equiv of Cu(OPiv)_2_, *x* equiv of HOPiv, 4 h, and 150 °C. Catalyst loading is relative to anisole per single Rh atom. Cu(OPiv)_2_ and HOPiv loading relative to a single Rh atom. Bold ratios are *o*/*m*/*p* ratios. HMB used as the internal standard. Error bars represent the standard deviation for a minimum of three independent reactions.

Previously, we reported that carboxylic acid concentration has two major effects on arene alkenylation: (1) there is an inverse first-order dependence on carboxylic acid concentration for the anaerobic conversion of benzene to styrene using **1** as the catalyst precursor and Cu(OPiv)_2_, which is consistent with reversible benzene C–H activation,^45^ and (2) the *m*/*p* selectivity of oxidative toluene alkenylation using ethylene as the olefin is dependent on the pivalic acid concentration.^54^ In the absence of pivalic acid, a 1:1 ratio of *m*/*p* alkenylated toluene products was observed, while at high pivalic acid concentrations, an approximate 2:1 *m*/*p* was observed. To assess if changing the pivalic acid equivalents will affect the *o*/*m*/*p* ratio of anisole alkenylation, similar to previous observations with toluene alkenylation, we varied the pivalic acid equivalents (relative to single Rh atom) at 0, 240, and 600 equiv (Figure 2). Without the addition of pivalic acid, we observed 43(3) total TOs of vinyl anisole products after 4 h with a 1:5:10 *o*/*m*/*p* selectivity. At these conditions, the amount of 2-methoxystyrene is minimal with a switch in selectivity from 3-methoxystyrene to 4-methoxystyrene compared to reactions with pivalic acid. Adding 600 equiv of pivalic acid to the reaction solution causes the reaction to slow relative to no pivalic acid with only 18(4) total TOs of methoxystyrenes after 4 h. The *o*/*m*/*p* selectivity is 1:3.6:1.3, which shows that the reaction is more selective for 3-methoxystyrene in the presence of pivalic acid, while the reaction is selective for 4-methoxystyrene in the absence of pivalic acid.

**Figure 3:**
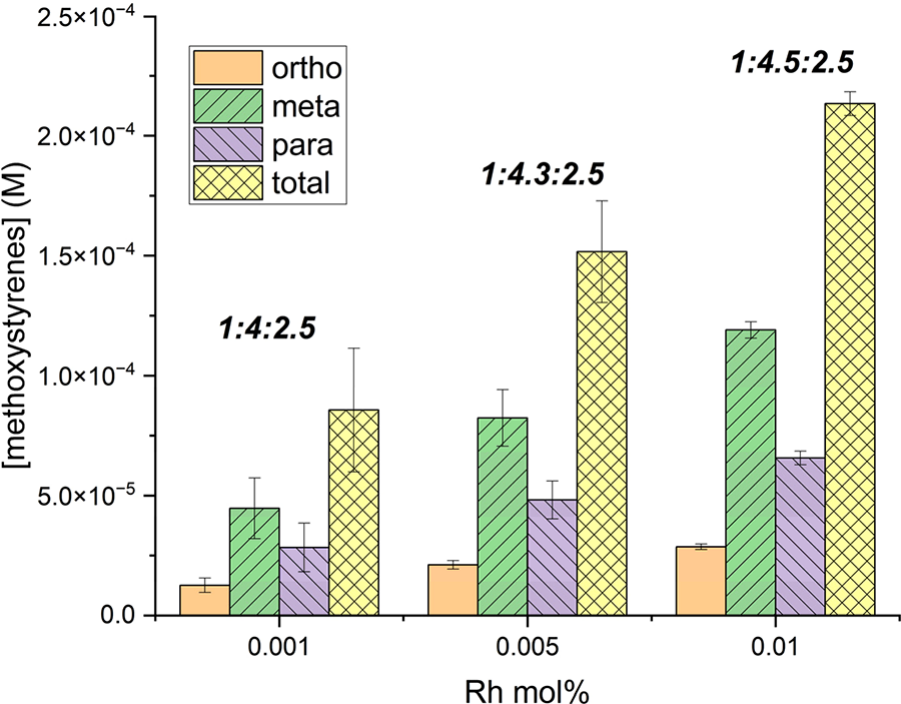
Catalyst loading optimization for oxidative anisole alkenylation catalyzed by [(η^2^-C_2_H_4_)_2_Rh(μ-OAc)]_2_. Reaction conditions: *x* mol % of [(η^2^-C_2_H_4_)_2_Rh(μ-OAc)]_2_, 7.5 mL of anisole, 50 psig of ethylene, 120 equiv of Cu(OPiv)_2_, 240 equiv of HOPiv, 4 h, and 150 °C. Catalyst loading is relative to anisole per single Rh atom. Cu(OPiv)_2_ and HOPiv loading relative to a single Rh atom. Bold ratios are *o*/*m*/*p* ratios. HMB used as the internal standard. Error bars represent the standard deviation for a minimum of three independent reactions.

Using 0.005 mol % of **1**, we observe less than a 2-fold decrease in total moles of alkenyl anisole product [44(6) TOs or 152 μmol] when compared to using 0.01 mol % of catalyst and no significant change in the *o*/*m*/*p* ratio (Figure 3). When the loading of **1** is lowered to 0.001 mol %, there was a greater than 2-fold decrease in moles of product after 4 h [124(37) TOs or 85.7 μmol] and no statistically significant change in the *o*/*m*/*p* selectivity compared to when using 0.01 mol % of Rh.

**Figure 4:**
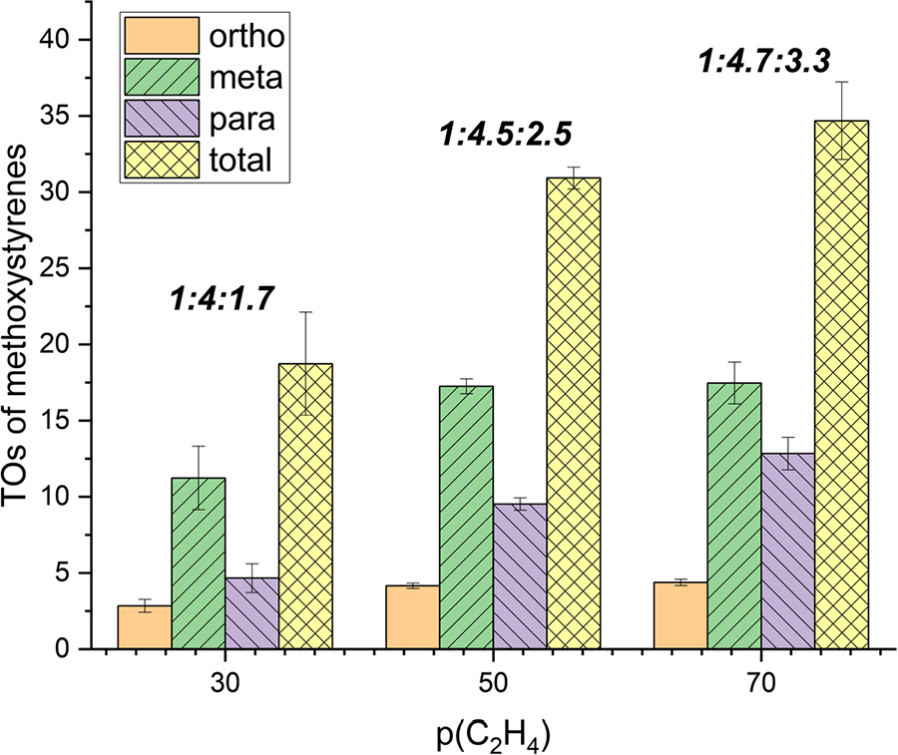
Ethylene pressure optimization for oxidative anisole alkenylation catalyzed by [(η^2^-C_2_H_4_)_2_Rh(μ-OAc)]_2_. Reaction conditions: 0.01 mol % of [(η^2^-C_2_H_4_)_2_Rh(μ-OAc)]_2_, 7.5 mL of anisole, *x* psig of ethylene, 120 equiv of Cu(OPiv)_2_, 240 equiv of HOPiv, 4 h, and 150 °C. Catalyst loading is relative to anisole per single Rh atom. Cu(OPiv)_2_ and HOPiv loading relative to a single Rh atom. Bold ratios are *o*/*m*/*p* ratios. HMB used as the internal standard. Error bars represent the standard deviation for a minimum of three independent reactions.

We studied the effect of varying the ethylene pressure (Figure 4). Decreasing the ethylene pressure from 50 to 30 psig gives 19(3) TOs of methoxystyrenes with an *o*/*m*/*p* ratio of 1:4:1.7, which is an increase in 3-methoxystyrene selectivity compared to reactions with 50 psig of ethylene. Increasing the ethylene pressure to 70 psig increased the TOs of product to 35(3) TOs of methoxystyrenes. There is a significant dependence of the *o*/*m*/*p* selectivity on the ethylene pressure. At 70 psig of ethylene, the *o*/*m*/*p* selectivity is 1:4.7:3.3, which is a decrease in selectivity for 3-methoxystyrene compared to the reaction at 50 psig of ethylene.

**Figure 5:**
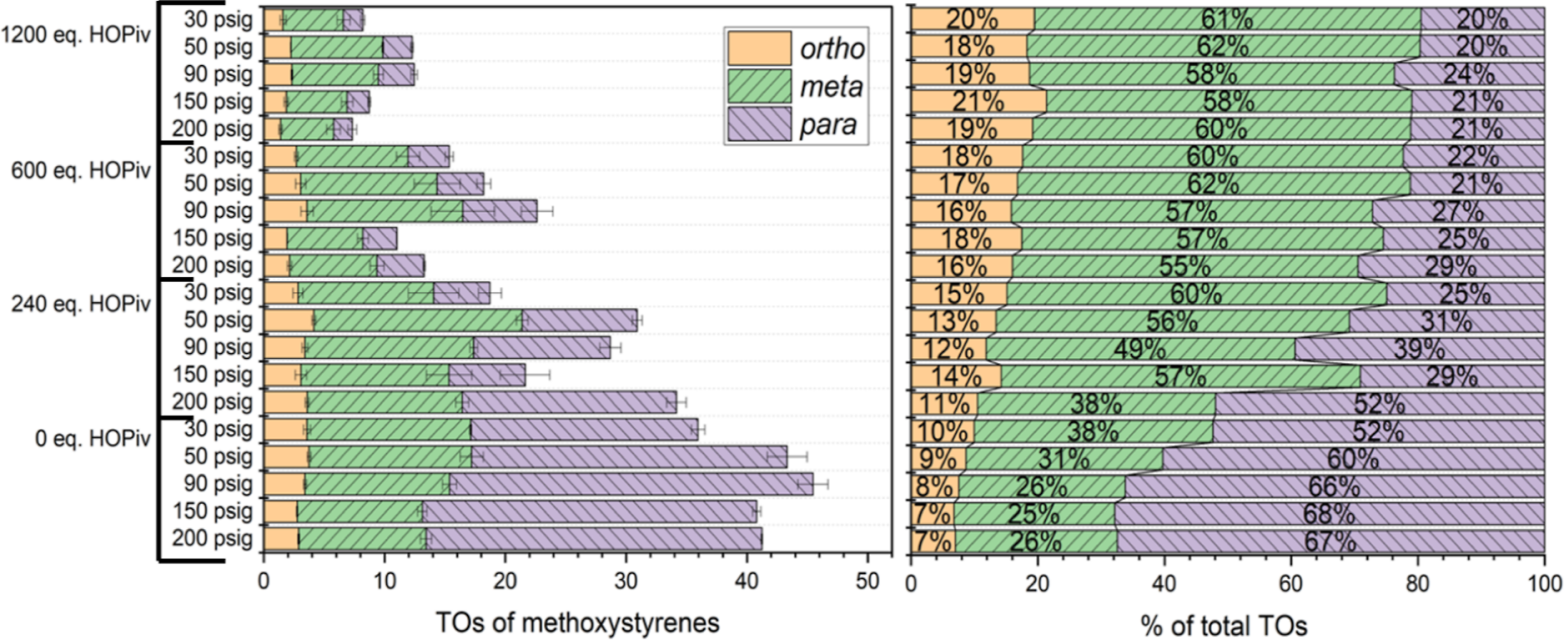
TOs of methoxystyrenes as a function of pivalic acid concentration and ethylene pressure (left) and % total TOs of the *ortho*, *meta*, and *para* regioisomers (right). Reaction conditions: 0.01 mol % of [(η^2^-C_2_H_4_)_2_Rh(μ-OAc)]_2_, 7.5 mL of anisole, *y* psig of ethylene, 120 equiv of Cu(OPiv)_2_, and *x* equiv of HOPiv. Catalyst loading is relative to anisole per single Rh atom. Cu(OPiv)_2_ and HOPiv loading relative to a single Rh atom. HMB used as the internal standard. Error bars represent the standard deviation for a minimum of three independent reactions. The decrease in total TOs at 150 and 200 psig of ethylene is a result of using a different reaction vessel better suited for higher pressures (see the Supporting Information for more information on reactor design).

To extend our selectivity studies, we probed anisole ethenylation using 5 different ethylene pressures (30, 50, 90, 150, and 200 psig) and 4 different pivalic acid equivalents (0, 240, 600, and 1200 equiv) (Figure 5). When no pivalic acid is added to the reaction (0 equiv of HOPiv) and the ethylene pressure is 200 psig (i.e., low [HOPiv] and high [C_2_H_4_]), the *o*/*m*/*p* ratio is 1:6:10, being selective for the 4-methoxystyrene product. When 240 equiv of pivalic acid is added to the reaction and the ethylene pressure is decreased from 200 to 30 psig, the *o*/*m*/*p* ratio changes from 1:3.5:5 to 1:3:1. When 600 and 1200 equiv of pivalic acid are added to the reaction, at all pressures of ethylene, the *o*/*m*/*p* ratio is statistically the same at 1:3:1.

**Figure 6:**
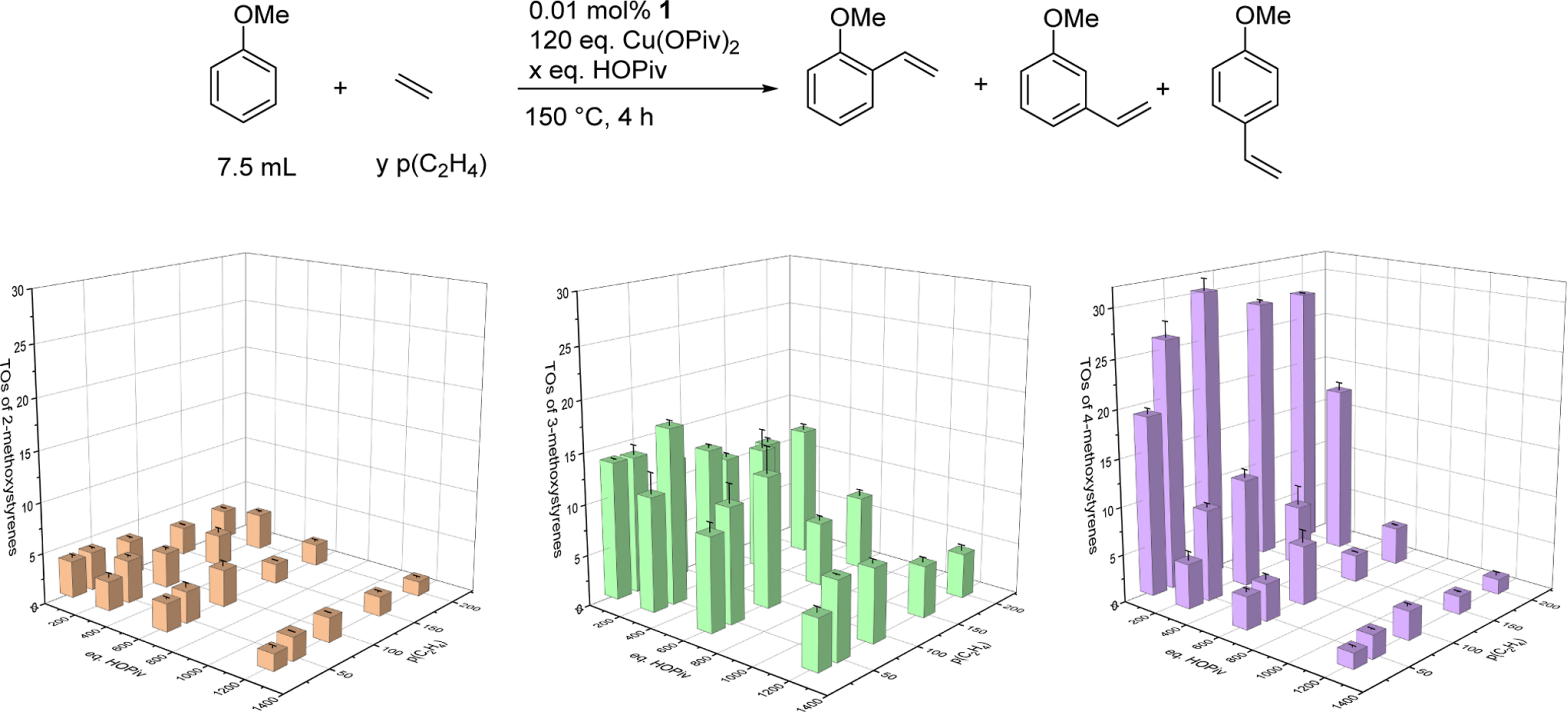
TOs of 2-methoxystyrene (left), 3-methoxystyrene (middle), and 4-methoxystyrene (right) as a function of pivalic acid concentration and ethylene pressure. Reaction conditions: 0.01 mol % of [(η^2^-C_2_H_4_)_2_Rh(μ-OAc)]_2_, 7.5 mL of anisole, *y* psig of ethylene, 120 equiv of Cu(II), and *x* equiv of HOPiv. Catalyst loading is relative to anisole per single Rh atom. Cu(OPiv)_2_ and HOPiv loading relative to a single Rh atom. HMB used as the internal standard. Error bars represent the standard deviation for a minimum of three independent reactions.

The TOs of 4-methoxystyrene seem to disproportionately decrease compared to 2- or 3-methoxystyrene with the addition of pivalic acid to the reaction (Figure 6). We performed several control experiments to probe whether the decrease in TOs of 4-methoxystyrene could be attributed to a side reaction with pivalic acid. First, we tested for the potential consumption of 4-methoxystyrene by performing the reaction in the absence of pivalic acid to completion, and then we monitored the amount of 4-methoxystyrene after the addition of 240 equiv of pivalic acid at several time points (Figure S11). The amount of 4-methoxystyrene stayed consistent at 155 μmol under reaction conditions in the absence and presence of pivalic acid, suggesting that there is no significant consumption of 4-methoxystyrene under catalytic reaction conditions.

Next, we performed analogous reactions using Pd(OAc)_2_ as the catalyst precursor. Pd(OAc)_2_ has been shown to be a catalyst for converting arenes and olefins in the presence of an oxidant to alkenyl arenes.^37−42^ We have demonstrated arene alkenylation using [(η^2^-C_2_H_4_)_2_Rh(μ-OAc)]_2_ and Pd(OAc)_2_ catalyst precursors in the presence of CuX_2_ (X = acetate, pivalate, and 2-ethylhexanoate) oxidants.^41^ Our studies of Rh and Pd catalyst precursors converting monosubstituted arenes (i.e., toluene, anisole, chlorobenzene, and trifluorotoluene) and olefins (i.e., ethylene, methyl acrylate, and styrene) to the corresponding alkenyl arene product showed approximately 2–3:1 *m*/*p* ratios when using [(η^2^-C_2_H_4_)_2_Rh(μ-OAc)]_2_ as the catalyst precursor for both electron-rich and electron-deficient arenes, while catalysis with Pd(OAc)_2_ as the catalyst precursor gave variable *o*/*m*/*p* ratios that were more sensitive to arene electronics than the Rh catalysis (i.e., approximate 2:2:1 for electronic-rich arenes and 1:3:1 for electron-deficient arenes). The above studies led us to conclude that the C–H activation step using Pd(OAc)_2_ exhibits a more electrophilic aromatic substitution character compared to the C–H activation step using [(η^2^-C_2_H_4_)_2_Rh(μ-OAc)]_2_.

**Figure 7:**
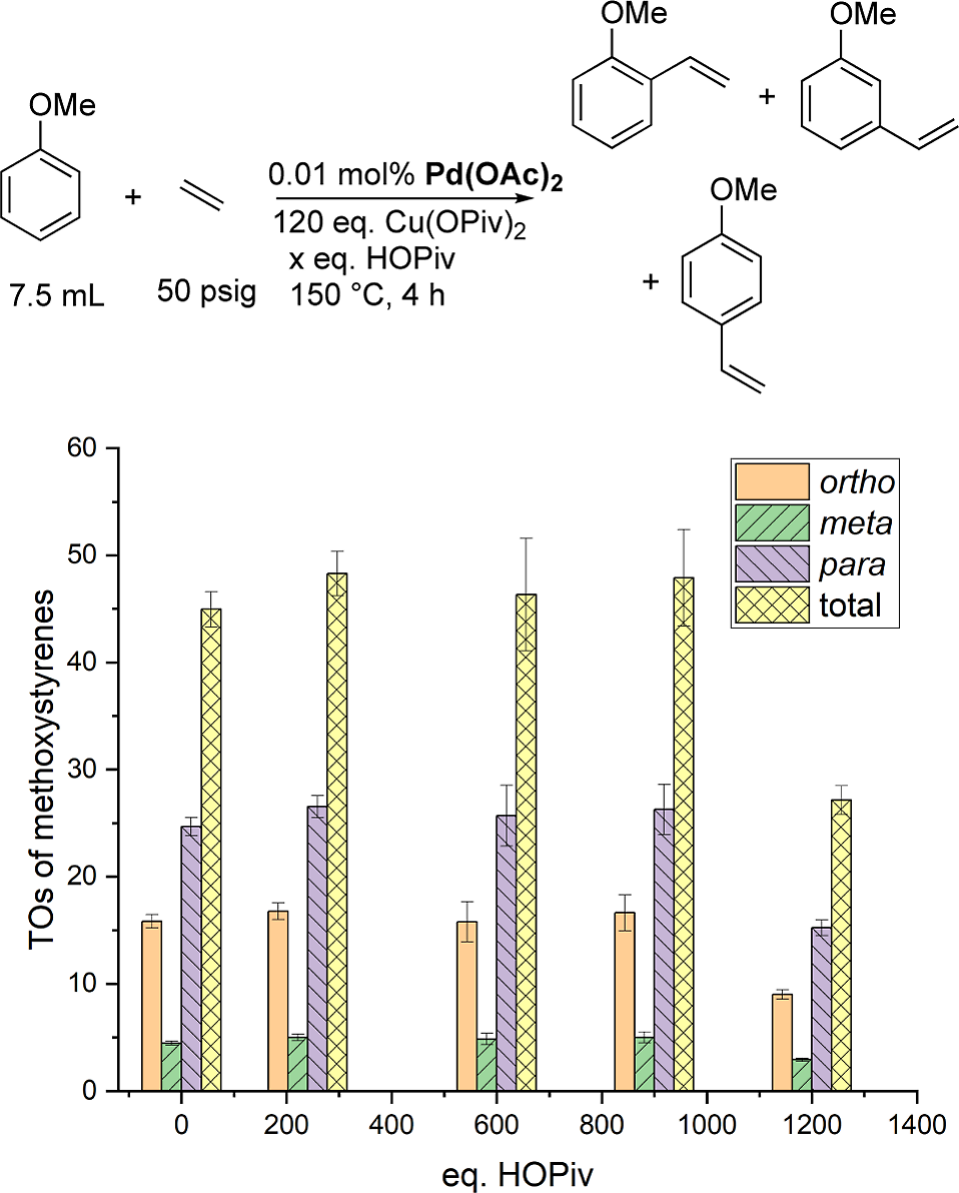
TOs of methoxystyrenes as a function of the pivalic acid concentration using Pd(OAc)_2_ as the catalyst precursor. Reaction conditions: 0.01 mol % of Pd(OAc)_2_, 7.5 mL of anisole, 50 psig of ethylene, 120 equiv of Cu(OPiv)_2_, and *x* equiv of HOPiv. Catalyst loading is relative to anisole per single Pd atom. Cu(OPiv)_2_ and HOPiv loading relative to a single Pd atom. HMB used as the internal standard. Error bars represent the standard deviation for a minimum of three independent reactions.

Figure 7 shows TOs of methoxystyrenes as a function of pivalic acid equivalents using 0.01 mol % of Pd(OAc)_2_ (relative to anisole), 120 equiv of Cu(OPiv)_2_ (relative to Pd), and 50 psig of ethylene at 150 °C for 4 h. The total TOs of methoxyarenes stay consistent at ∼45(2) TOs for 4 of the pivalic acid equivalents tested (0, 240, 600, and 900 equiv). The total TOs of methoxystyrenes drops to 27(1) when 1200 equiv of pivalic acid is added. The *o*/*m*/*p* ratio stays consistent at 1:0.3:1.7 across all pivalic acid equivalents tested for Pd catalysis. Thus, the observed changes in the *o*/*m*/*p* ratio as a function of pivalic acid concentration observed for Rh catalysis are not observed for Pd catalysis.

**Scheme 4:**
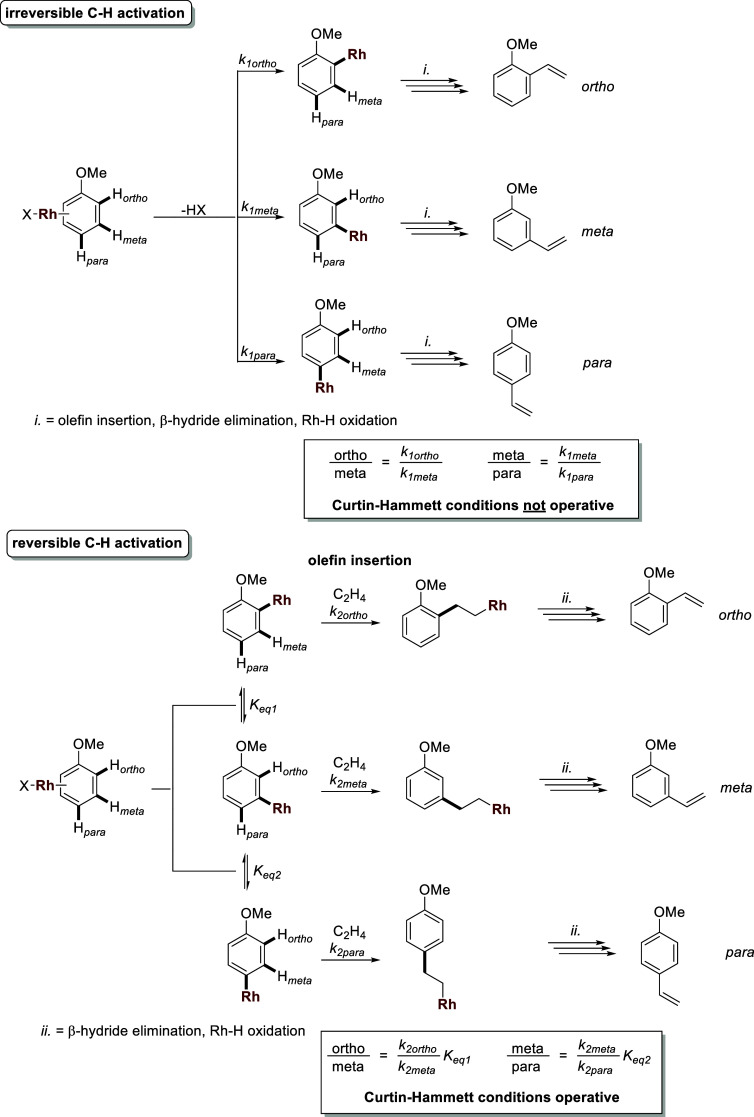
Factors that Affect the *o*/*m*/*p* Regioselectivity of Anisole Alkenylation in the Limiting Cases of Fully Irreversible and Reversible C–H Activation Steps

For Rh catalysis, it is apparent that the *o*/*m*/*p* regioselectivity of anisole alkenylation is dependent on the ethylene pressure and pivalic acid equivalents. For transition-metal-mediated C(sp^2^)–H functionalization reactions, the C–H activation step to form metal-aryl intermediates can dictate the regioselectivity of the functionalization reaction. That is, in the limiting case of irreversible C–H activation, the *o*/*m*/*p* regioselectivity will be dictated solely by the relative rates of C–H activation of the *ortho*, *meta*, and *para* C–H bonds (i.e., *k*_1ortho_ vs *k*_1meta_ vs *k*_1para_ in Scheme 4). Note that, for simplicity, here, the *k*_1_ values include access to each C–H bond (i.e., an inability to access an ortho C–H bond is included in *k*_1ortho_). Conversely, in the limiting case of fully reversible C–H activation before an irreversible olefin insertion step, Curtin–Hammett conditions would be operative. That is, both the equilibrium constants (i.e., *K*_eq 1_ and *K*_eq 2_) and the rate of olefin insertion (i.e., *k*_2ortho_ vs *k*_2meta_ vs *k*_2para_) will dictate the *o*/*m*/*p* regioselectivity (Scheme 4).

**Scheme 5:**
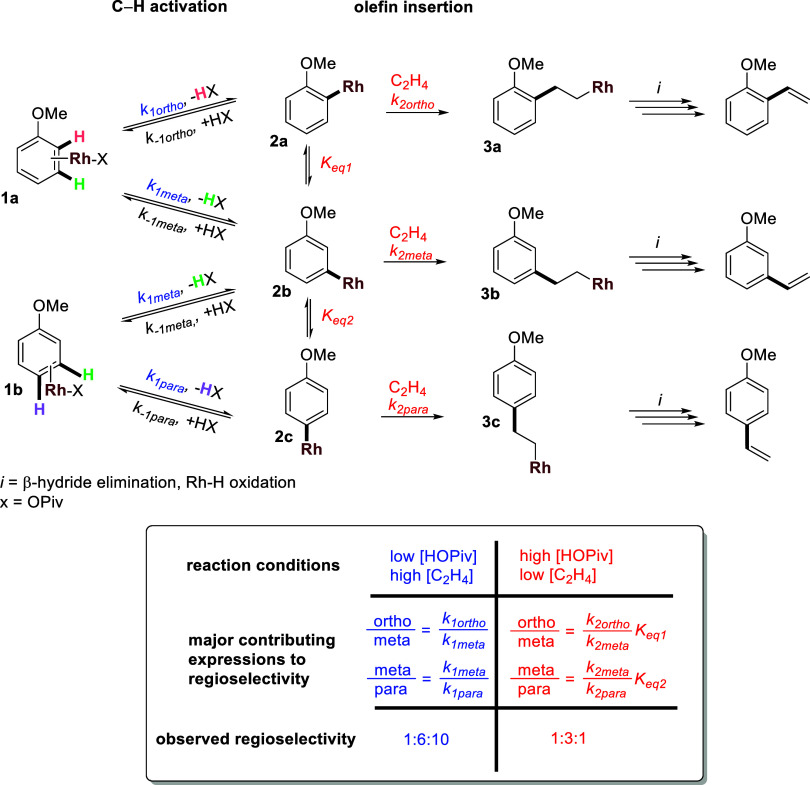
Abbreviated Mechanism for Rh-Catalyzed Anisole Ethenylation (X = OPiv) and Summary of Regioselectivities

We propose that anisole C–H activation is at least partially reversible for Rh-catalyzed oxidative anisole alkenylation, which is consistent with the inverse first-order dependence on carboxylic acid concentration observed for benzene C–H activation in a previous study.^45^ The regioselectivity of the C–H functionalization is dictated, at least in part, by the equilibria of Rh–aryl intermediates and the relative rates of olefin insertion. In Scheme 5, the active Rh species (generically depicted as Rh–X) can bind to anisole η^2^ at the 2,3-position or the 3,4-position (note: we assume that η^2^ coordination at the 1,2 position of anisole is sterically blocked). Intermediates **1a** and **1b** can undergo C–H activation at the *ortho*, *meta*, or *para* C–H bonds to form Rh–aryl intermediates **2a**, **2b**, and **2c**. Here, for simplicity, the forward and reverse C–H activation steps are depicted as a single step; however, previous DFT calculations suggest that reversible benzene C–H activation by Rh(I) to form Rh–aryl intermediate occurs in a stepwise C–H oxidative addition and O–H reductive coupling rather than a concerted metalation–deprotonation.^53^ Rh–aryl intermediates **2a** and **2b** can interconvert (*K*_eq 1_), and **2b** and **2c** can interconvert (*K*_eq 2_). Although equilibrium might not be fully achieved under catalytic conditions, C–H activation is likely reversible (i.e., kinetically competitive with olefin insertion). Intermediates **2a**, **2b**, and **2c** can undergo olefin insertion to form **3a**, **3b**, and **3c** intermediates. Subsequent β-hydride elimination and Rh–H oxidation by Cu(OPiv)_2_ yields the 2-, 3-, or 4-methoxystyrene products. We proposed that olefin insertion is likely irreversible.

Our studies indicate that *o*/*m*/*p* selectivity varies with the pivalic acid concentration and ethylene concentrations. These results are consistent with *k*_–1_ competing with *k*_2_. As shown in Scheme 5, increasing the concentration of pivalic acid (HX in Scheme 5) should increase the rate of reverse C–H activation (*k*_–1_[HX]) and allow equilibration between intermediates **2a**, **2b**, and **2c** that is competitive with olefin insertion. In contrast, increasing the concentration of ethylene should increase the rate of olefin insertion (*k*_2_[C_2_H_4_]) to irreversibly form intermediates **3a**, **3b**, and **3c**. Thus, at low pivalic acid concentrations and high ethylene concentrations, it is anticipated that the reverse of C–H activation is relatively slow (the *k*_–1_[HX] term decreases) compared to olefin insertion, and the *o*/*m*/*p* regioselectivity will be dictated, at the extreme, by the relative rates of C–H activation (*k*_1ortho_ vs *k*_1meta_ vs *k*_1para_) to form intermediates **2a**, **2b**, and **2c**. For reactions with 0 equiv of pivalic acid and 200 psig of ethylene pressure, we observe an approximate 1:6:10 *o*/*m*/*p* regioselectivity. This is consistent with a lower barrier for *para* C–H activation to form the *para* intermediate (**2c**) relative to *ortho* and *meta* C–H activation (i.e., *k*_1para_ > *k*_1ortho_, *k*_1meta_). Conversely, at high pivalic acid concentrations and low ethylene concentrations, the reverse C–H activation is likely relatively fast (the *k*_–1_[HX] term increases), and the olefin insertion step is relatively slow (the *k*_2_[C_2_H_4_] term decreases). In this kinetic regime, the *o*/*m*/*p* regioselectivity would be dictated, at the extreme, by the equilibria constants between **2a**, **2b**, and **2c** and the rates of olefin insertion for each (*k*_2ortho_, *k*_2meta_, and *k*_2para_). We expect a 2:2:1 *o*/*m*/*p* ratio if all C–H bonds of anisole were equally accessible, and there is no thermodynamic advantage to activating one over the others. Reactions with 1200 equiv of pivalic acid and 30 psig of ethylene pressure, the observed *o*/*m*/*p* ratio is 1:3:1, indicating that either there is a slight preference for the *meta* intermediate **2b** to undergo olefin insertion relative to **2a** and **2c** and/or there is a difference in equilibria for **2a**, **2b**, and **2c**.

**Figure 8:**
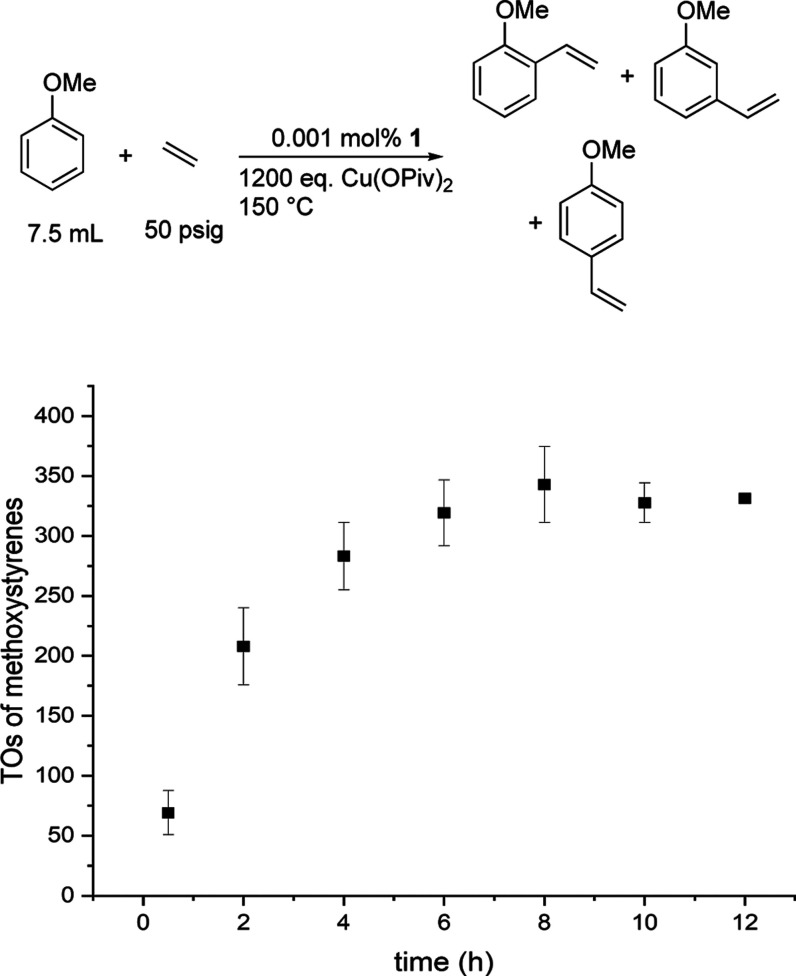
TOs vs time plot for oxidative anisole alkenylation using [(η^2^-C_2_H_4_)_2_Rh(μ-OAc)]_2_ as the catalyst precursor. Reaction conditions: 0.001 mol % of [(η^2^-C_2_H_4_)_2_Rh(μ-OAc)]_2_, 7.5 mL of anisole, 50 psig of ethylene, and 1200 equiv of Cu(II). Catalyst loading is relative to anisole per single Rh atom. Cu(OPiv)_2_ loading relative to a single Rh atom. HMB used as the internal standard. Error bars represent the standard deviation for a minimum of three independent reactions.

We studied the longevity of Rh catalysis using 1200 equiv of Cu(OPiv)_2_. Assuming that 2 equiv of Cu(OPiv)_2_ is consumed per TO of methoxystyrene produced, the maximum TO using 1200 equiv of Cu(OPiv)_2_ under anaerobic conditions is 600 TO. Heating a solution of neat anisole with the rhodium catalyst precursor [(η^2^-C_2_H_4_)_2_Rh(μ-OAc)]_2_ (0.001 mol % relative to anisole) in the presence of Cu(OPiv)_2_ (1200 equiv relative to a single Rh atom), we observe catalysis up to 8 h with 343(32) TOs of methoxystyrenes with a 1:5:13 *o*/*m*/*p* ratio (Figure 8). After 8 h, the TOs plateau at ∼57% yield (based on the 1200 equiv of Cu(OPiv)_2_ as the limiting reagent), indicating some form of decomposition/deactivation of either the Cu(II) oxidant or the Rh catalyst or product inhibition. Cu(OPiv)_2_ (Cu^2+^) is blue, and Cu(OPiv) (Cu^1+^) is bronze. Hence, we can qualitatively assess based on color that Cu(OPiv)_2_ in solution has not been completely consumed to form Cu(OPiv) (Figure S13).

**Figure 9:**
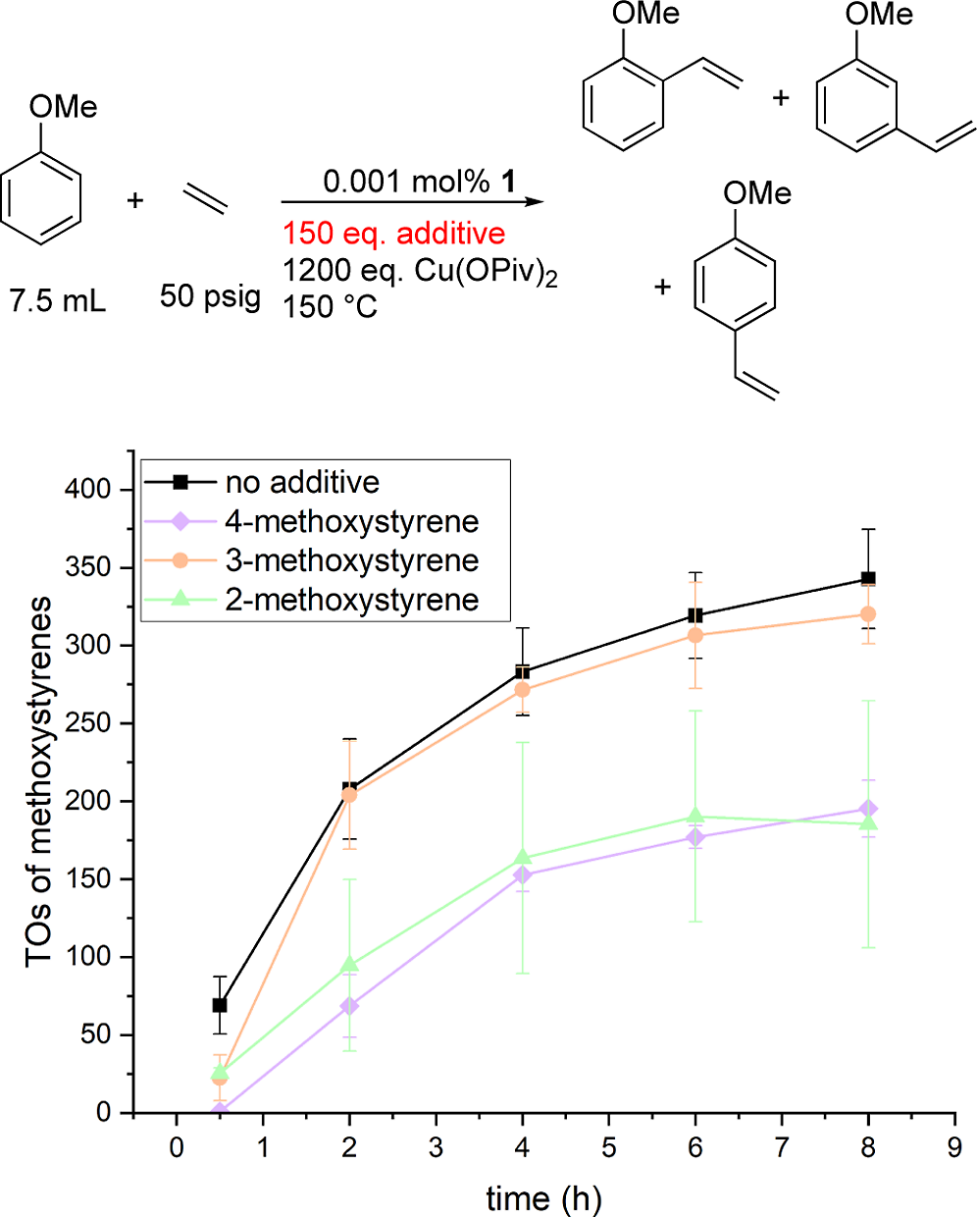
TOs vs time plot for oxidative anisole alkenylation using [(η^2^-C_2_H_4_)_2_Rh(μ-OAc)]_2_ as a catalyst precursor testing for product inhibition. Reaction conditions: 0.001 mol % of [(η^2^-C_2_H_4_)_2_Rh(μ-OAc)]_2_, 7.5 mL of anisole, 50 psig of ethylene, and 1200 equiv of Cu(OPiv)_2_. 150 equiv of additive. Catalyst loading is relative to anisole per single Rh atom. Cu(OPiv)_2_ and additive loading relative to a single Rh atom. HMB used as the internal standard. TOs were adjusted to account for methoxystyrene being used as an additive in the reaction. Error bars represent the standard deviation for a minimum of three independent reactions.

We performed studies investigating catalyst longevity by testing if any of the methoxystyrene products inhibit catalysis. Using [(η^2^-C_2_H_4_)_2_Rh(μ-OAc)]_2_ (0.001 mol % relative to anisole) and 1200 equiv of Cu(OPiv)_2_ (relative to a single Rh atom), we added 150 equiv of 2-, 3-, and 4-methoxystyrene (relative to a single Rh atom) into separate reactions and monitored the TOs versus time (Figure 9).

**Scheme 6:**
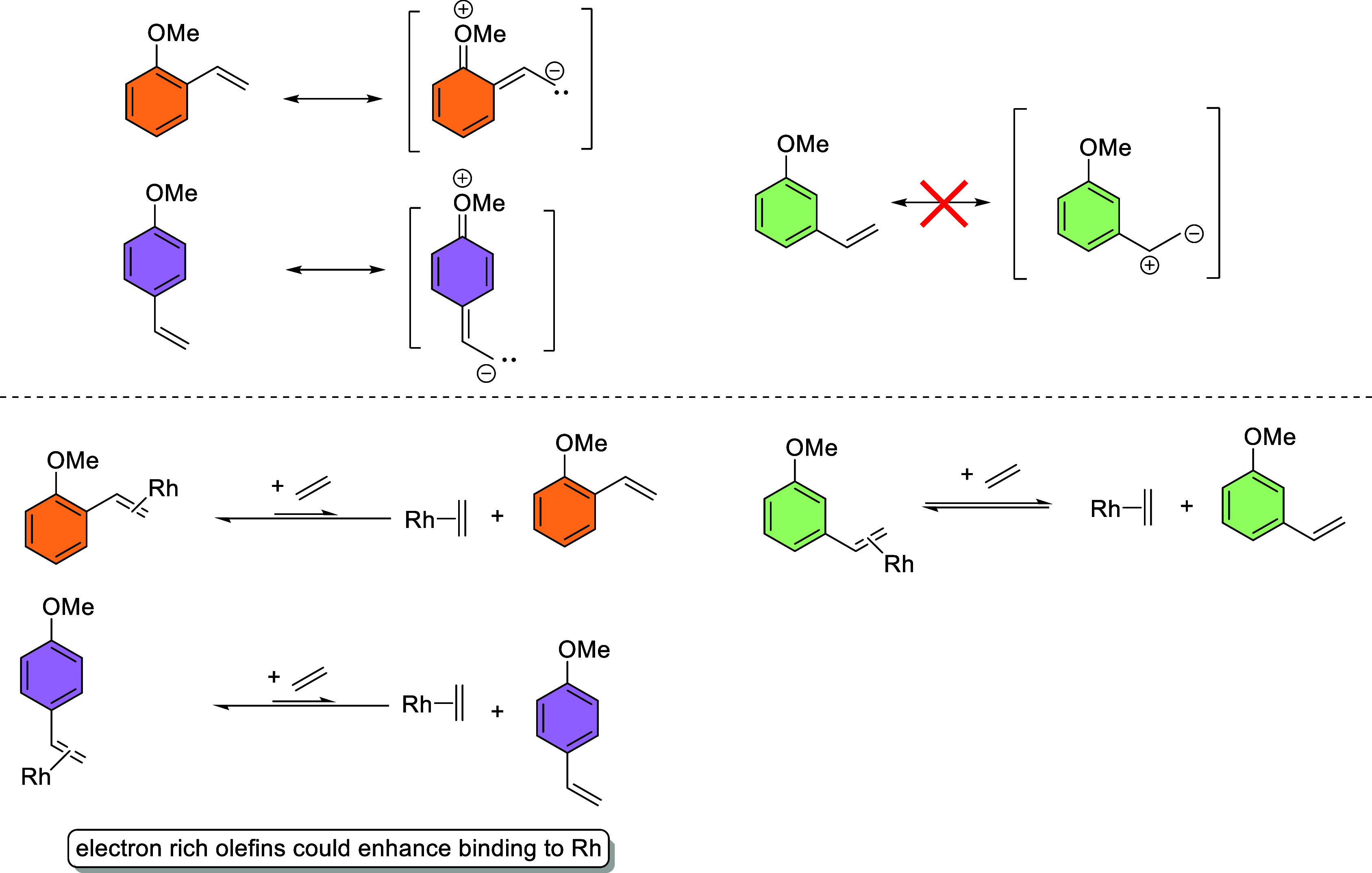
Potential Explanation for Catalysis Inhibition by 2-Methoxystyrene and 4-Methoxystyrene

The addition of 150 equiv of 3-methoxystyrene does not inhibit the rate of catalysis as TOs vs time is statistically identical to catalysis without any 3-methoxystyrene added. The addition of 150 equiv of 2- or 4-methoxystyrene lowers the yield of methoxystyrenes [based on the Cu(OPiv)_2_ amount] to approximately 29% compared with catalysis without added methoxystyrene after 8 h and, thus, does inhibit the catalytic activity. The catalysis is inhibited by the *ortho* and *para* isomers of methoxystyrene, and thus, catalyst productivity is inherently limited in batch reactors for which the products cannot be separated from the reaction mixture. The inhibition by the *ortho* and *para* products suggests an electronic effect. The partial negative charge on the olefin that is present for the *ortho* and *para* isomers likely enhances the binding of olefin to the active Rh species and would inhibit Rh catalysis (Scheme 6). The *meta* isomer does not have a similar electronic effect and therefore would not inhibit catalysis, which is consistent with our findings.

**Scheme 7:**
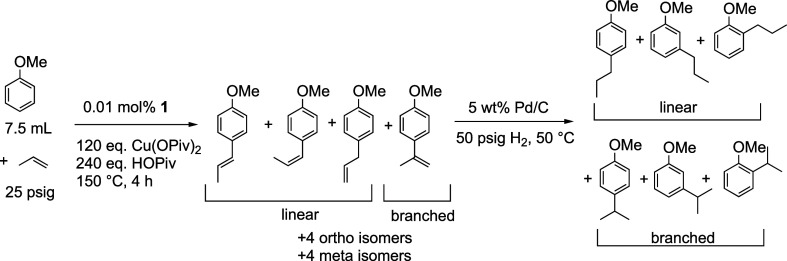
Rh-Catalyzed Anisole Alkenylation Using Propylene as the Olefin and Subsequent Hydrogenation to Yield 6 Products

**Figure 10:**
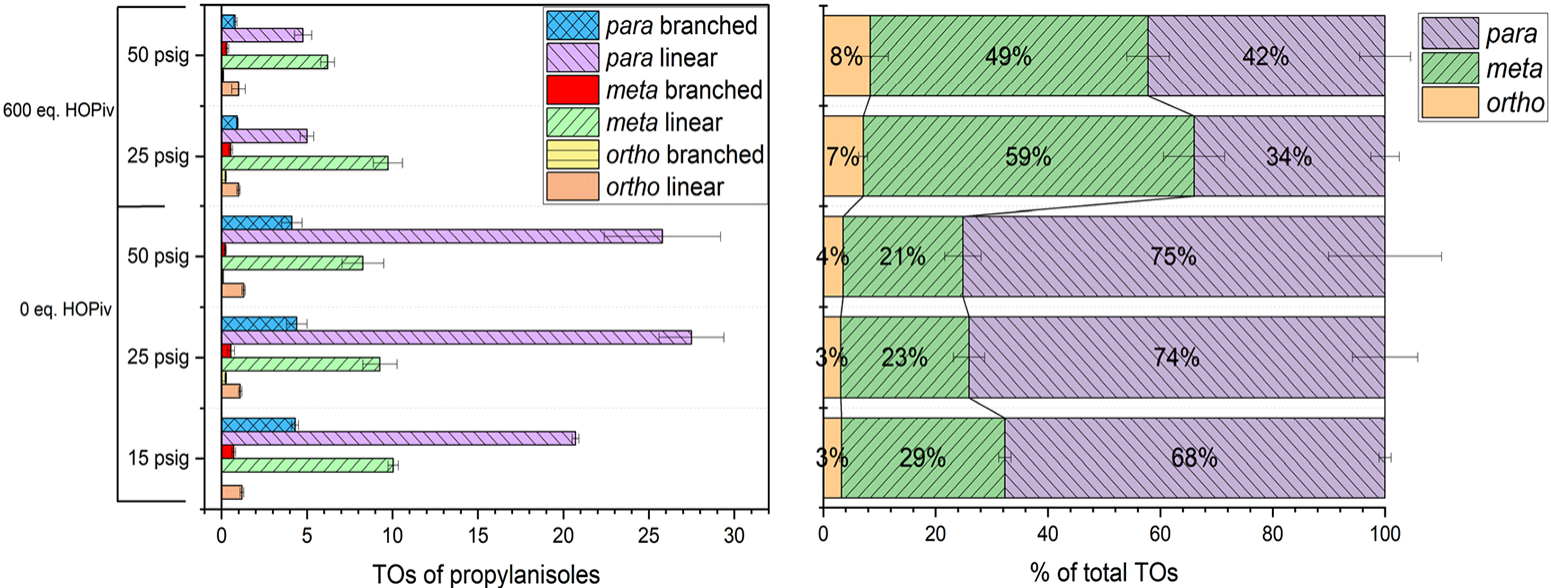
TOs of propylanisoles from oxidative anisole propenylation using [(η^2^-C_2_H_4_)_2_Rh(μ-OAc)]_2_ as a catalyst precursor, followed by hydrogenation with Pd/C (left) and % total TOs of the *ortho*, *meta*, and *para* regioisomers (right). Reaction conditions for oxidative anisole alkenylation: 0.01 mol % of [(η^2^-C_2_H_4_)_2_Rh(μ-OAc)]_2_, 7.5 mL of anisole, *x* psig of propylene, *y* equiv of HOPiv, and 120 equiv of Cu(OPiv)_2_. Catalyst loading is relative to anisole per single Rh atom. Cu(OPiv)_2_ and HOPiv loading relative to a single Rh atom. HMB used as the internal standard. Reaction conditions for hydrogenation: 100 mg of 5 wt % Pd/C, 7.5 mL of ethanol, 50 psig of H_2_, 50 °C, and 18 h. Error bars represent the standard deviation for a minimum of three independent reactions.

We sought to probe the selectivity for the reaction with propylene as the olefin. The alkenylation using α-olefins produces both the Markovnikov (branched) and anti-Markovnikov (linear) products (Scheme 7). Using propylene as the olefin, there are 12 propenylanisoles (vide supra) as products (Scheme 7). Similar to ethylene, there are *ortho*, *meta*, and *para* isomers. Also, there are 3 linear products (i.e., anti-Markovnikov) possible: *E* or *Z* methoxyprop-1-enylbenzenes and allyl anisole. There is 1 branched product (i.e., Markovnikov) possible, methoxyprop-2-enylbenzene. To ease product analysis, we hydrogenated the mixture of 12 propenylanisole isomers to yield 6 propylanisoles (1 linear and 1 branched for each *ortho*, *meta*, and *para* isomer), which are all identifiable (Figure S7). On testing 3 different propylene pressures (15, 25, and 50 psig) and 2 pivalic acid equivalents (0 and 600 equiv), we observed similar trends compared to catalysis using ethylene as the olefin (Figure 10). At 0 equiv of pivalic acid, the *o*/*m*/*p* regioselectivity is 1:6:20. At 600 equiv of pivalic acid, the *o*/*m*/*p* regioselectivity changes from 1:8.5:5 at 25 psig of propylene to 1:6:5 at 50 psig propylene. When propylene is the olefin, less *ortho* product is made compared with reactions with ethylene. For example, the sum of *ortho* branched and linear products is <10% of the total TOs for all reaction conditions tested with propylene, whereas analogous reactions with ethylene give an *ortho* product at 10–20% of the total TOs. We hypothesize that the added steric hindrance of inserting propylene into the Rh–aryl bond ortho to the methoxy substituent is kinetically unfavorable. Similar to the catalysis using ethylene as the olefin, we hypothesize that the *o*/*m*/*p* regioselectivity for propylene is under Curtin–Hammett conditions. At low pivalic acid concentrations (0 equiv) and high propylene pressures (50 psig), a 1:6:20 *o*/*m*/*p* ratio is observed, which is consistent with a kinetic preference for *para* C–H bond activation over the *ortho* and *meta*. At high pivalic acid concentrations and low propylene pressures, we observe a 1:8.5:5 ratio, which is consistent with a slight preference for olefin insertion to form the *para* alkenylated product (a 2:2:1 *o*/*m*/*p* ratio would be expected with no kinetic or thermodynamic preferences). Reaction conditions that favor production of *para*-alkenylated products (0 equiv of pivalic acid) yield approximately 24(1)% selectivity for *trans*-anethole [24(1)% of total products is *trans*-anethole], whereas reaction conditions that do not favor the production of *para*-alkenylated products (600 equiv of pivalic acid) yield approximately 15(1)% selectivity for *trans*-anethole (Table 1). Similar to using ethylene as the olefin, the addition of pivalic acid to the reaction disproportionately affects the production of *para*-alkenylated products, which we attribute to the Curtin–Hammett control of regioselectivity.

**Table 1:** Tabulated Data from Figure 10

entry	equiv of HOPiv	*p*(C_3_H_6_) (psig)	total TOs	selectivity for *trans*-anethole (%)	*o*/*m*/*p* ratio
1	0	15	37(1)	24(0)	1:8:20
2	0	25	42(4)	25(1)	1:7.5:25
3	0	50	38(4)	24(1)	1:6:20
4	600	25	17(1)	12(1)	1:8.5:5
5	600	50	12(1)	17(1)	1:6:5

**Scheme 8:**
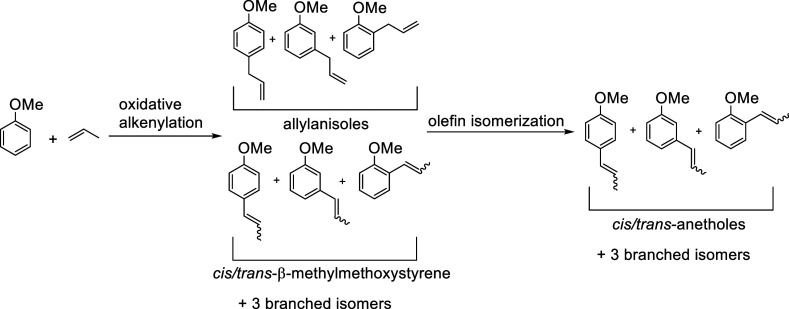
Anisole Alkenylation Using Propylene as the Olefin and Subsequent Olefin Isomerization to Yield 9 Products

**Scheme 9:**
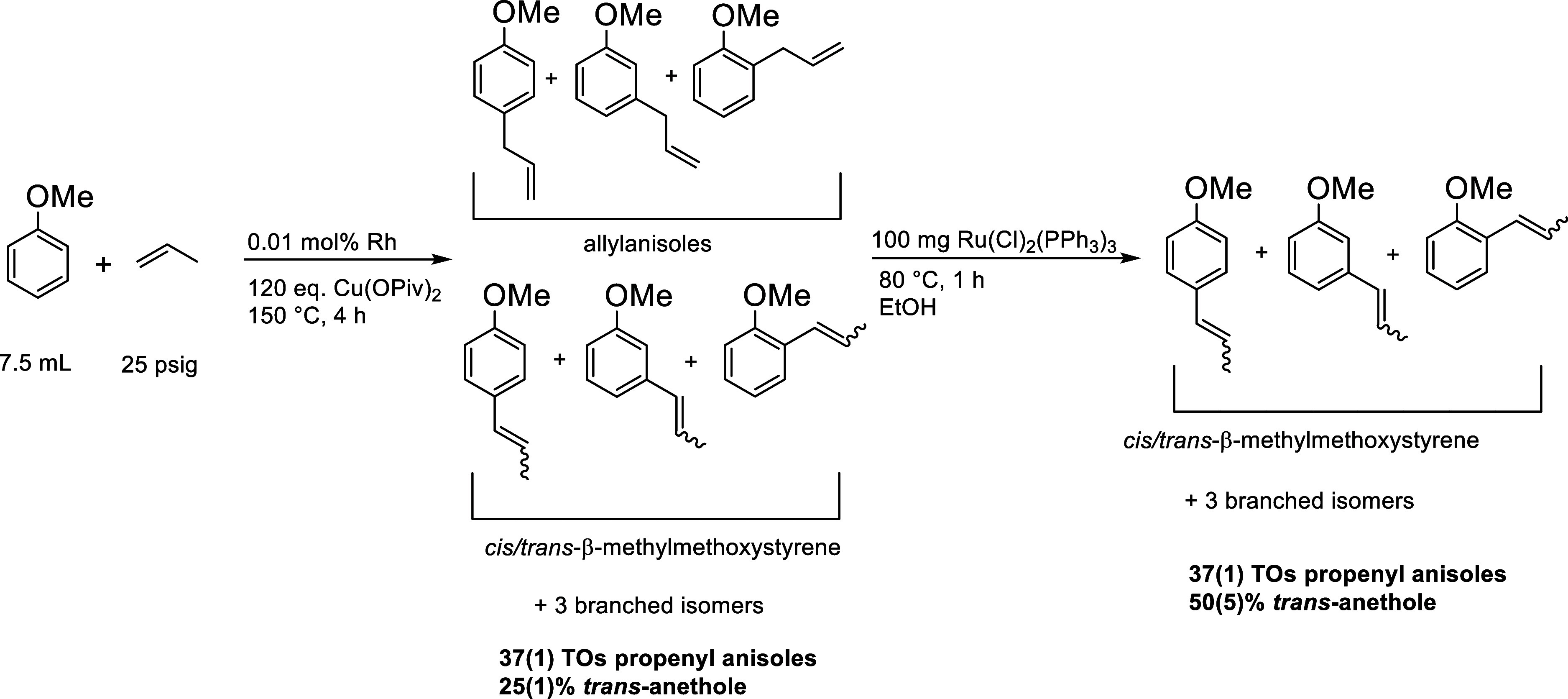
Rh-Catalyzed Anisole Alkenylation Using Propylene as the Olefin and Subsequent Olefin Isomerization Catalyzed by Ru(Cl)_2_(PPh_3_)_3_ to Yield 50(5)% *trans*-Anethole

We sought to use an olefin isomerization catalyst to isomerize the allylanisole products to their respective *cis*- and *trans*-β-methylmethoxystyrenes to increase the selectivity for *trans*-anethole (Scheme 8). The isomerization of 4-allylanisole to *cis* and *trans*-anethole using Ru-based catalysts has previously been reported.^55^ We envisioned a process for which we could first perform the Rh-catalyzed anisole alkenylation with propylene under conditions for the highest selectivity for *para* alkenylated products (no pivalic acid, 25 psig of propylene) and then add Ru(Cl)_2_(PPh_3_)_3_ as an olefin isomerization catalyst to increase the yield of anethole products. Rh-catalyzed (0.01 mol % of Rh relative to anisole) anisole alkenylation with 25 psig of propylene as the olefin and no pivalic acid yields approximately 37(1) TOs of propenyl anisoles with 24(0)% of TOs being *trans*-anethole after 4 h at 150 °C. Ru(Cl)_2_(PPh_3_)_3_ (100 mg) was added to the reaction mixture with 7.5 mL of ethanol and heated at 80 °C for 1 h. Analysis of the resulting mixture showed 37(1) TOs of propenyl anisoles with an increased yield of 50(5)% for *trans*-anethole (Scheme 9 and Figure S15).

**Figure 11:**
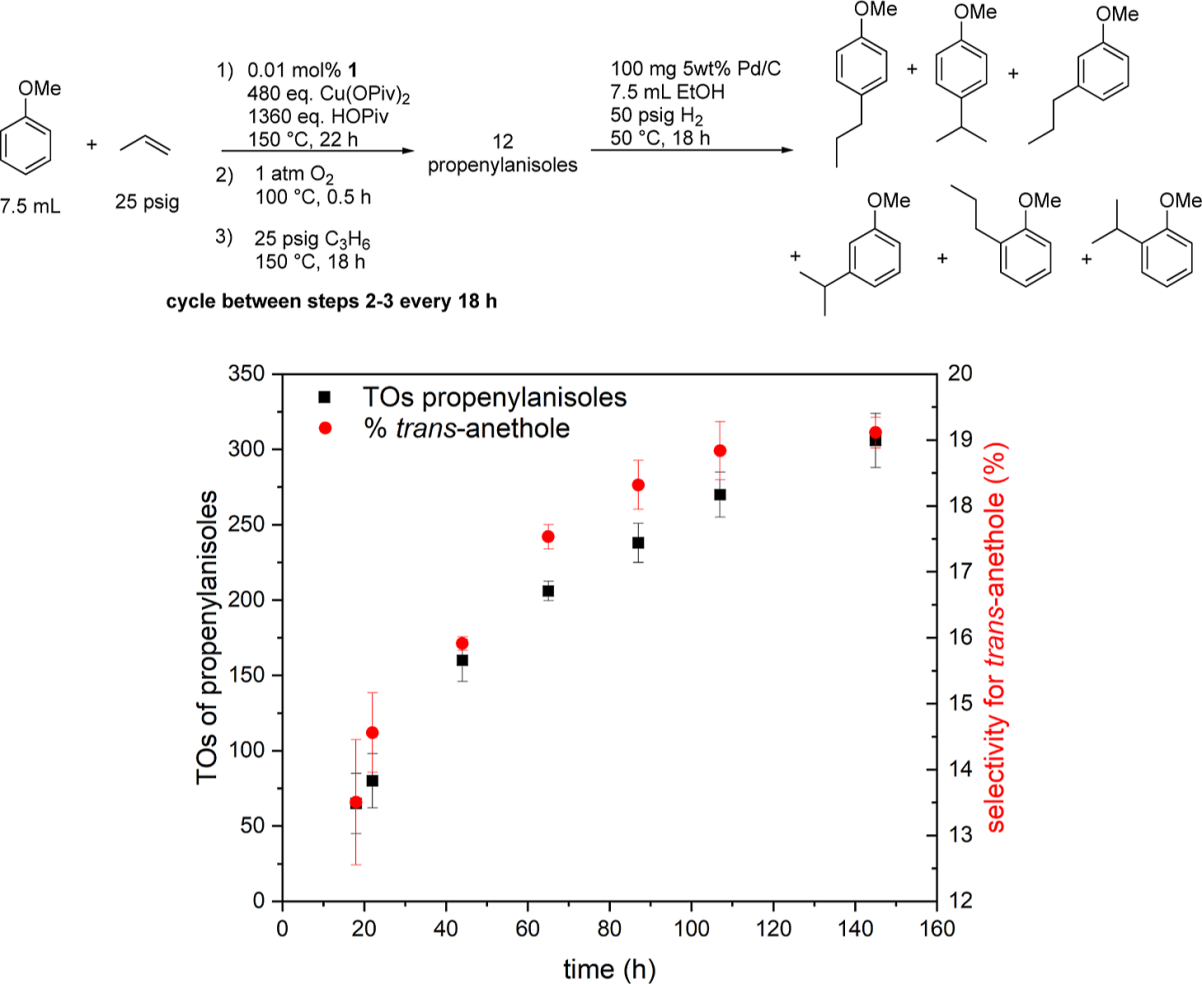
TOs of propenylanisoles from oxidative anisole alkenylation using [(η^2^-C_2_H_4_)_2_Rh(μ-OAc)]_2_ as a catalyst precursor and in situ O_2_ recycling of Cu(OPiv)_2_. Reaction conditions for oxidative anisole alkenylation: 0.01 mol % of [(η^2^-C_2_H_4_)_2_Rh(μ-OAc)]_2_, 7.5 mL of anisole, 25 psig of propylene, 1360 equiv of HOPiv, and 480 equiv of Cu(OPiv)_2_. Catalyst loading is relative to anisole per single Rh atom. Cu(OPiv)_2_ and HOPiv loading relative to a single Rh atom. HMB used as the internal standard. Reaction conditions for Cu(OPiv)_2_ recycling: propylene atmosphere is purged out under N_2_ flow; then, 1 atm O_2_ is charged into the reaction and allowed to stir at 100 °C for 0.5 h. Error bars represent the standard deviation for a minimum of three independent reactions.

Using 480 equiv of Cu(OPiv)_2_, the theoretical yield under anaerobic conditions is 240 TOs of propenylanisoles. The advantage of using Cu(OPiv)_2_ (or other Cu(II) carboxylate salts) as an in situ oxidant is that the stoichiometric amount of Cu(OPiv) produced from the reaction can then be regenerated back to Cu(OPiv)_2_ by using air or O_2_. The use of air-recyclable Cu(II) salts (i.e., CuCl_2_) as an oxidant have been demonstrated to be viable on an industrial scale by the Pd-catalyzed Wacker–Hoechst process for ethylene oxidation.^56−58^ We demonstrated that we can reach higher than 100% yield of propenylanisoles based on Cu(OPiv)_2_ by performing O_2_ recycling every 18–20 h (Figure 11). We first charge the reaction mixture of 0.01 mol % of Rh (relative to anisole), 480 equiv of Cu(OPiv)_2_, and 1360 equiv of pivalic acid (relative to a single Rh atom) with 25 psig of propylene and heated at 150 °C for 22 h. Next, the reactor is charged with 1 atm O_2_ with stirring at 100 °C for 0.5 h or until a color change from bronze/colorless to blue (Figure S13). After the O_2_ recycling step, the dioxygen atmosphere is removed, and the reaction is charged with 25 psig of propylene. After 6 O_2_ recycling steps, 306(18) TOs of propenylanisoles were achieved after 145 h. The selectivity for *trans*-anethole increases from 14(1) to 19(0)%. The approximate 5% increase in *trans*-anethole selectivity over the course of the reaction under aerobic conditions is different from the selectivity of *trans*-anethole when the reaction is performed anaerobically. This could suggest that the incorporation of O_2_ during the recycling step impacts the catalyst speciation and increases the selectivity for *trans*-anethole. Previously, for Ir-catalyzed arene propenylation, we have demonstrated that catalyst speciation likely changes during the reaction with a significant impact on anti-Markovnikov to Markovnikov selectivity.^36^ The *o*/*m*/*p* regioselectivity at the end of the reaction is 1:8.5:5. Due to the complexity of the product analysis, we can only report on the *o*/*m*/*p* regioselectivity at the end of the reaction. A similar reaction with 1 atm of O_2_ directly added into the reactors was successful (Figure S16) but is not directly compared to the above because the use of O_2_ and hydrocarbon mixtures requires a different reactor design with additional safety precautions.

## Summary and Conclusions

We have demonstrated the direct alkenylation of anisole to produce alkenyl anisoles with [(η^2^-C_2_H_4_)_2_Rh(μ-OAc)]_2_ as the catalyst precursor and Cu(OPiv)_2_ as the in situ oxidant. Controlling selectivity for the *para* alkenylated product versus the *meta* alkenylated product is possible, varying between 1:6:10 and 1:3:1 *o*/*m*/*p* ratio when ethylene is the olefin depending on reaction conditions. When propylene is the olefin, the *o*/*m*/*p* regioselectivity varies between 1:8:20 and 1:8.5:5. The addition of 2- or 4-methoxystyrene inhibits the rate of catalysis and affects the overall longevity of catalysis, which suggests that electron-rich olefins might have enhanced binding to the active Rh catalyst.

## Experimental Section

### General Considerations

Unless otherwise noted, all reactions were carried out under an inert atmosphere in a N_2_-filled glovebox. Glovebox purity was maintained by periodic N_2_ purges to maintain a dioxygen concentration <20 ppm. Ethylene and propylene (99.9%) were purchased in gas cylinders from Linde Gas and Equipment and used as received. Anisole (99%) was purchased from Beantown Chemical and degassed by bubbling with N_2_ for 30 min before being stored in a N_2_-filled glovebox. Di-μ-acetatotetrakis(dihaptoethene)dirhodium(I) (**1**) was synthesized according to a previously published procedure.^59^ Cu(II) pivalate was synthesized according to a previously published procedure.^60^ The material 5 wt % Pd/C (unreduced) was purchased from Acros Chemicals and used as received. Tris(triphenylphosphine)ruthenium(II)dichloride was purchased from Sigma-Aldrich and used as received. Gas chromatography/mass spectrometry (GC–MS) was performed using a Shimadzu GCMS-QP2020 NX instrument with a 30 m × 0.25 mm Rxi-5 ms column with a 0.25 μm film thickness using electron impact ionization. Gas chromatography–flame ionization detector (GC–FID) was performed using a Shimadzu GC-2014 instrument with a 30 mm × 0.32 mm DB-5 ms UI column with a 0.25 μm film thickness. For the GC–FID instrument, TOs were quantified by linear regression analysis of chromatograms using the authentic product or estimated using compounds of similar molecular weights and composition. Plots of peak area versus molar ratio gave regression lines relative to those of internal standard hexamethylbenzene. Slopes and correlation coefficients for the following compounds are as follows: 2-methoxystyrene (1.77, 0.9939), 3-methoxystyrene (1.50, 0.9978), 4-methoxystyrene (1.62, 0.9971), and *trans*-anethole (1.35, 0.9999). TOs of propenyl and propylanisoles were estimated using the slope for *trans*-anethole.

### Representative Procedure for Oxidative Anisole Alkenylation

A 10 mL stock solution of [(η^2^-C_2_H_4_)_2_Rh(μ-OAc)]_2_ (15.1 mg, 6.9 μmol, 1 equiv per Rh atom) was prepared in anisole. To oven-dried 3-oz. Astraglass innovations Fisher-Porter reactors attached with adjustable pressure poppet check valves for pressure safety release, 1 mL of stock solution, Cu(OPiv)_2_ (220 mg, 828 μmol, 120 equiv per Rh atom), HOPiv (169 mg, 1.65 mmol, 240 equiv per Rh atom), HMB (2.24 mg, 13.8 μmol, 2 equiv per Rh atom), and 6.5 mL of anisole (total 7.5 mL reaction solution) were added. The Fisher-Porter reactors were sealed and removed from the glovebox. Ethylene (50 psig) was added to each reactor by using a high-pressure gas manifold. Reactors were stirred and heated at 150 °C using a silicone oil bath hot plate for 4 h. A polycarbonate blast shield (4.7 mm thickness, 30 in. height) was placed in front of the stirring reactors during heating. After cooling to room temperature, an aliquot of this solution was diluted with benzene and washed with a saturated solution of NaHCO_3_ prior to GC–FID analysis.

### Representative Procedure for Longevity Study of Oxidative Anisole Alkenylation

A 10 mL stock solution of [(η^2^-C_2_H_4_)_2_Rh(μ-OAc)]_2_ (1.51 mg, 0.69 μmol, 1 equiv per Rh atom) was prepared in anisole. To oven-dried 3-oz. Astraglass Innovations Fisher-Porter reactors attached with adjustable pressure poppet check valves for pressure safety release, 1 mL of stock solution, Cu(OPiv)_2_ (220 mg, 828 μmol, 1200 equiv per Rh atom), HMB (22.4 mg, 138 μmol, 20 equiv per Rh atom), and 6.5 mL of anisole (total 7.5 mL reaction solution) were added. The Fisher-Porter reactors were sealed and taken out of the glovebox. The appropriate amount of 2-, 3-, and 4-methoxystyrene (13.8 mg, 104 μmol, 150 equiv per Rh atom) was added to the reactor under dinitrogen flow on a Schlenk line. Ethylene (50 psig) was added to each reactor using a high-pressure gas manifold. Reactors were stirred and heated at 150 °C using a silicone oil bath hot plate. A polycarbonate blast shield (4.7 mm thickness and 30″ height) was placed in front of the stirring reactors during heating. After 0.5 h and subsequently every 2 h, the reactors were allowed to cool under dinitrogen flow, and an aliquot of the reaction mixture was taken, diluted with benzene, and washed with a saturated solution of NaHCO_3_ prior to GC–FID analysis. After taking the aliquot, the reactors were charged with ethylene (50 psig) and heated at 150 °C until the next time point.

### Representative Procedure for Oxidative Anisole Alkenylation Using Propylene as the Olefin

A 10 mL stock solution of [(η^2^-C_2_H_4_)_2_Rh(μ-OAc)]_2_ (15.1 mg, 6.9 μmol, 1 equiv per Rh atom) was prepared in anisole. To oven-dried 3-oz. Astraglass Innovations Fisher-Porter reactors attached with adjustable pressure poppet check valves for pressure safety release, 1 mL of stock solution, Cu(OPiv)_2_ (220 mg, 828 μmol, 120 equiv per Rh atom), HOPiv (169 mg, 1.65 mmol, 240 equiv per Rh atom), HMB (2.24 mg, 13.8 μmol, 2 equiv per Rh atom), and 6.5 mL of anisole (total 7.5 mL reaction solution) were added. The Fisher-Porter reactors were sealed and taken out of the glovebox. Propylene (25 psig) was added to each reactor by using a high-pressure gas manifold. Reactors were stirred and heated at 150 °C using a silicone oil bath hot plate for 4 h. A polycarbonate blast shield (4.7 mm thickness and 30″ height) was placed in front of the stirring reactors during heating. After cooling to room temperature, an aliquot of this solution was diluted with benzene and washed with a saturated solution of NaHCO_3_ prior to GC–FID analysis.

### Representative Procedure for Hydrogenation Using Pd/C

After performing the oxidative anisole alkenylation using propylene as the olefin, the reaction mixture was hydrogenated for ease of product analysis. The reaction mixture used for oxidative anisole alkenylation was directly added to 7.5 mL of ethanol and 100 mg of 5 wt % Pd/C. To avoid potential mixtures of air and H_2_, the reactors were first purged of the air atmosphere and filled with N_2_; then, the N_2_ atmosphere was evacuated and H_2_ (50 psig) was added to each reactor using a high-pressure gas manifold. Reactors were stirred and heated at 50 °C using a silicone oil bath hot plate for 18 h. A polycarbonate blast shield (4.7 mm thickness, 30″ height) was placed in front of the stirring reactors during heating. After the mixture was cooled to room temperature, an aliquot of this solution was diluted with benzene and washed with a saturated solution of NaHCO_3_, prior to GC–FID analysis.

### Representative Procedure for Isomerization Using Ru(Cl)_2_(PPh_3_)_3_

The reaction mixture used for oxidative anisole alkenylation was directly added to 7.5 mL of ethanol and 100 mg of Ru(Cl)_2_(PPh_3_)_3_. The reaction mixture was stirred and heated at 80 °C for 18 h using a silicone oil bath. A polycarbonate blast shield (4.7 mm thickness and 30″ height) was placed in front of the stirring reactors during heating. After cooling to room temperature, an aliquot of this solution was diluted with benzene and washed with a saturated solution of NaHCO_3,_ prior to GC–FID analysis.

## Supporting Information Available

GC–FID chromatograms, additional experimental details, materials, and photographs of the experimental setup (PDF)

## References

[ref1] Fahlbusch K.-G.; Hammerschmidt F.-J.; Panten J.; Pickenhagen W.; Schatkowski D.; Bauer K.; Garbe D.; Surburg H. Flavors and Fragrances. In Ullmann’s Encyclopedia of Industrial Chemistry; Wiley, 2003; pp 1–127.

[ref2] Aprotosoaie A. C.; Costache I.-I.; Miron A. Anethole and Its Role in Chronic Diseases. In Drug Discovery from Mother Nature; Gupta S. C., Prasad S., Aggarwal B. B., Eds.; Springer International Publishing, 2016; pp 247–267. 10.1007/978-3-319-41342-6_11 27771928

[ref3] Choo E. J.; Rhee Y.-H.; Jeong S.-J.; Lee H.-J.; Kim H. S.; Ko H. S.; Kim J.-H.; Kwon T.-R.; Jung J. H.; Kim J. H.; et al. Anethole Exerts Antimetatstaic Activity via Inhibition of Matrix Metalloproteinase 2/9 and AKT/Mitogen-Activated Kinase/Nuclear Factor Kappa B Signaling Pathways. Biol. Pharm. Bull. 2011, 34 (1), 41–46. 10.1248/bpb.34.41. 21212515

[ref4] Ha B.; Ko H.; Kim B.; Sohn E. J.; Jung J. H.; Kim J. S.; Yoon J. J.; Won G.; Kim J.-H.; Jung D.-b.; et al. Regulation of Crosstalk between Epithelial to Mesenchymal Transition Molecules and MMP-9 Mediates the Antimetastatic Activity of Anethole in DU145 Prostate Cancer Cells. J.Nat. Products 2014, 77 (1), 63–69. 10.1021/np4006376. 24328151

[ref5] Ponte E. L.; Sousa P. L.; Rocha M. V. A. P.; Soares P. M. G.; Coelho-de-Souza A. N.; Leal-Cardoso J. H.; Assreuy A. M. S. Comparative study of the anti-edematogenic effects of anethole and estragole. Pharmacol. Rep. 2012, 64 (4), 984–990. 10.1016/S1734-1140(12)70895-2. 23087152

[ref6] Galicka A.; Krętowski R.; Nazaruk J.; Cechowska-Pasko M. Anethole prevents hydrogen peroxide-induced apoptosis and collagen metabolism alterations in human skin fibroblasts. Mol. Cell. Biochem. 2014, 394 (1–2), 217–224. 10.1007/s11010-014-2097-0. 24898780 PMC4118036

[ref7] Orav A.; Raal A.; Arak E. Essential oil composition of Pimpinella anisum L. fruits from various European countries. Nat. Prod. Res. 2008, 22 (3), 227–232. 10.1080/14786410701424667. 18266152

[ref8] Pereira C. G.; Meireles M. A. A. Economic analysis of rosemary, fennel and anise essential oils obtained by supercritical fluid extraction. Flavour Fragrance J. 2007, 22 (5), 407–413. 10.1002/ffj.1813.

[ref9] Dzamic A.; Sokovic M.; Ristic M. S.; Grijic-Jovanovic S.; Vukojevic J.; Marin P. D. Chemical composition and antifungal activity of Illicium verum and Eugenia caryophyllata essential oils. Chem. Nat. Compd. 2009, 45 (2), 259–261. 10.1007/s10600-009-9283-4.

[ref10] Zhang H.; Quek Z. J.; Jaenicke S.; Chuah G.-K. Hydrophobicity and co-solvent effects on Meerwein-Ponndorf-Verley reduction/dehydration cascade reactions over Zr-zeolite catalysts. J. Catal. 2021, 400, 50–61. 10.1016/j.jcat.2021.05.011.

[ref11] Liu Y.; Li M.; Liu T.; Tan J.; Rokhum S. L.; Zhang H.; Yang S.; Li H. Hydrophobic species-enabled acid-base multi-catalysis for stereoselective access to renewable trans-anethole. Dalton Trans. 2022, 51 (43), 16668–16680. 10.1039/D2DT02502G. 36278834

[ref12] Tan J.; Wu H.; Huang J.; Jian Y.; Zhang L.-L.; Zhang H.; Li H.; Yang S. Cascade upgrading of bio-based 4’-methoxypropiophenone to anethole enabled by hot-compressed alcohol over a Hf-phytic acid coordination catalyst. J. Supercrit. Fluids 2022, 189, 105696 10.1016/j.supflu.2022.105696.

[ref13] Zhang H.; Cheng F.; Li Y.; He C.; Li H.; Yang S. Polymeric organophosphate-hafnium unconventional MOFs nanohybrids enable high-efficiency upgrading of biomass feedstocks via cascade catalytic transfer hydrogenation-dehydration. Ind. Crops Prod. 2022, 188, 115606 10.1016/j.indcrop.2022.115606.

[ref14] Chen L.; Liu Y.; Zhang H.; Li Y.; Zhang S.; Hu Y.; Li H.; Yang S. Domino” synthesis of bio-derived anethole over facile-prepared hafnium phosphonate frameworks with efficient bifunctional acid sites. Reaction Chem. Eng. 2023, 8 (6), 1464–1475. 10.1039/D3RE00096F.

[ref15] Lail M.; Arrowood B. N.; Gunnoe T. B. Addition of Arenes to Ethylene and Propene Catalyzed by Ruthenium. J. Am. Chem. Soc. 2003, 125 (25), 7506–7507. 10.1021/ja035076k. 12812477

[ref16] Lail M.; Bell C. M.; Conner D.; Cundari T. R.; Gunnoe T. B.; Petersen J. L. Experimental and Computational Studies of Ruthenium(II)-Catalyzed Addition of Arene C-H Bonds to Olefins. Organometallics 2004, 23 (21), 5007–5020. 10.1021/om049404g.

[ref17] Foley N. A.; Lail M.; Gunnoe T. B.; Cundari T. R.; Boyle P. D.; Petersen J. L. Combined Experimental and Computational Study of TpRu{P(pyr)3}(NCMe)Me (pyr = N-pyrrolyl): Inter- and Intramolecular Activation of C-H Bonds and the Impact of Sterics on Catalytic Hydroarylation of Olefins. Organometallics 2007, 26 (23), 5507–5516. 10.1021/om700666y.

[ref18] Foley N. A.; Lail M.; Lee J. P.; Gunnoe T. B.; Cundari T. R.; Petersen J. L. Comparative Reactivity of TpRu(L)(NCMe)Ph (L = CO or PMe3): Impact of Ancillary Ligand L on Activation of Carbon-Hydrogen Bonds Including Catalytic Hydroarylation and Hydrovinylation/Oligomerization of Ethylene. J. Am. Chem. Soc. 2007, 129 (21), 6765–6781. 10.1021/ja068542p. 17488072

[ref19] Foley N. A.; Ke Z.; Gunnoe T. B.; Cundari T. R.; Petersen J. L. Aromatic C-H Activation and Catalytic Hydrophenylation of Ethylene by TpRu{P(OCH2)3CEt}(NCMe)Ph. Organometallics 2008, 27 (13), 3007–3017. 10.1021/om800275b.

[ref20] Foley N. A.; Lee J. P.; Ke Z.; Gunnoe T. B.; Cundari T. R. Ru(II) Catalysts Supported by Hydridotris(pyrazolyl)borate for the Hydroarylation of Olefins: Reaction Scope, Mechanistic Studies, and Guides for the Development of Improved Catalysts. Acc. Chem. Res. 2009, 42 (5), 585–597. 10.1021/ar800183j. 19296659

[ref21] Andreatta J. R.; McKeown B. A.; Gunnoe T. B. Transition metal catalyzed hydroarylation of olefins using unactivated substrates: Recent developments and challenges. J. Organomet. Chem. 2011, 696 (1), 305–315. 10.1016/j.jorganchem.2010.09.030.

[ref22] Joslin E. E.; McMullin C. L.; Gunnoe T. B.; Cundari T. R.; Sabat M.; Myers W. H. Catalytic Hydroarylation of Ethylene Using TpRu(L)(NCMe)Ph (L = 2,6,7-Trioxa-1-phosphabicyclo[2,2,1]heptane): Comparison to TpRu(L′)(NCMe)Ph Systems (L′ = CO, PMe3, P(pyr)3, or P(OCH2)3CEt). Organometallics 2012, 31 (19), 6851–6860. 10.1021/om300676e.

[ref23] Burgess S. A.; Joslin E. E.; Gunnoe T. B.; Cundari T. R.; Sabat M.; Myers W. H. Hydrophenylation of ethylene using a cationic Ru(ii) catalyst: comparison to a neutral Ru(ii) catalyst. Chem. Sci. 2014, 5 (11), 4355–4366. 10.1039/C4SC01665C.

[ref24] Periana R. A.; Liu X. Y.; Bhalla G. Novel bis-acac-O,O-Ir(iii) catalyst for anti-Markovnikov, hydroarylation of olefins operates by arene CH activation. Chem. Commun. 2002, 3000–3001. 10.1039/B208680H. 12536786

[ref25] Bhalla G.; Oxgaard J.; Goddard W. A.; Periana R. A. Anti-Markovnikov Hydroarylation of Unactivated Olefins Catalyzed by a Bis-tropolonato Iridium(III) Organometallic Complex. Organometallics 2005, 24 (13), 3229–3232. 10.1021/om0501733.

[ref26] McKeown B. A.; Foley N. A.; Lee J. P.; Gunnoe T. B. Hydroarylation of Unactivated Olefins Catalyzed by Platinum(II) Complexes. Organometallics 2008, 27 (16), 4031–4033. 10.1021/om8006008.

[ref27] McKeown B. A.; Gonzalez H. E.; Friedfeld M. R.; Gunnoe T. B.; Cundari T. R.; Sabat M. Mechanistic Studies of Ethylene Hydrophenylation Catalyzed by Bipyridyl Pt(II) Complexes. J. Am. Chem. Soc. 2011, 133 (47), 19131–19152. 10.1021/ja206064v. 22060179

[ref28] McKeown B. A.; Gonzalez H. E.; Michaelos T.; Gunnoe T. B.; Cundari T. R.; Crabtree R. H.; Sabat M. Control of Olefin Hydroarylation Catalysis via a Sterically and Electronically Flexible Platinum(II) Catalyst Scaffold. Organometallics 2013, 32 (14), 3903–3913. 10.1021/om400390e.

[ref29] McKeown B. A.; Gonzalez H. E.; Gunnoe T. B.; Cundari T. R.; Sabat M. PtII-Catalyzed Ethylene Hydrophenylation: Influence of Dipyridyl Chelate Ring Size on Catalyst Activity and Longevity. ACS Catal. 2013, 3 (6), 1165–1171. 10.1021/cs400231f.

[ref30] McKeown B. A.; Gonzalez H. E.; Friedfeld M. R.; Brosnahan A. M.; Gunnoe T. B.; Cundari T. R.; Sabat M. Platinum(II)-Catalyzed Ethylene Hydrophenylation: Switching Selectivity between Alkyl- and Vinylbenzene Production. Organometallics 2013, 32 (9), 2857–2865. 10.1021/om400306w.

[ref31] Luedtke A. T.; Goldberg K. I. Intermolecular Hydroarylation of Unactivated Olefins Catalyzed by Homogeneous Platinum Complexes. Angew. Chem., Int. Ed. 2008, 47 (40), 7694–7696. 10.1002/anie.200800524. 18726981

[ref32] Clement M. L.; Grice K. A.; Luedtke A. T.; Kaminsky W.; Goldberg K. I. Platinum(II) Olefin Hydroarylation Catalysts: Tuning Selectivity for the anti-Markovnikov Product. Chem.—Eur. J. 2014, 20 (52), 17287–17291. 10.1002/chem.201405174. 25377546

[ref33] Bair J. S.; Schramm Y.; Sergeev A. G.; Clot E.; Eisenstein O.; Hartwig J. F. Linear-Selective Hydroarylation of Unactivated Terminal and Internal Olefins with Trifluoromethyl-Substituted Arenes. J. Am. Chem. Soc. 2014, 136 (38), 13098–13101. 10.1021/ja505579f. 25171744

[ref34] Saper N. I.; Ohgi A.; Small D. W.; Semba K.; Nakao Y.; Hartwig J. F. Nickel-catalysed anti-Markovnikov hydroarylation of unactivated alkenes with unactivated arenes facilitated by non-covalent interactions. Nat. Chem. 2020, 12 (3), 276–283. 10.1038/s41557-019-0409-4. 32042137 PMC11723504

[ref35] Weissman H.; Song X.; Milstein D. Ru-Catalyzed Oxidative Coupling of Arenes with Olefins Using O2. J. Am. Chem. Soc. 2001, 123 (2), 337–338. 10.1021/ja003361n. 11456523

[ref36] Ketcham H.; Zhu W.; Gunnoe T. B. Highly Anti-Markovnikov Selective Oxidative Arene Alkenylation Using Ir(I) Catalyst Precursors and Cu(II) Carboxylates. Organometallics 2024, 43 (7), 774–786. 10.1021/acs.organomet.4c00030. 38606203 PMC11005047

[ref37] Fujiwara Y.; Moritani I.; Danno S.; Asano R.; Teranishi S. Aromatic substitution of olefins. VI. Arylation of olefins with palladium(II) acetate. J. Am. Chem. Soc. 1969, 91 (25), 7166–7169. 10.1021/ja01053a047. 27462934

[ref38] Yamada T.; Sakakura A.; Sakaguchi S.; Obora Y.; Ishii Y. Oxidative arylation of ethylene with benzene catalyzed by Pd(OAc)2/heteropoly acid/O2 system. Nouv. J. Chem. 2008, 32 (4), 738–742. 10.1039/b716425d.

[ref39] Kubota A.; Emmert M. H.; Sanford M. S. Pyridine Ligands as Promoters in PdII/0-Catalyzed C-H Olefination Reactions. Org. Lett. 2012, 14 (7), 1760–1763. 10.1021/ol300281p. 22409653

[ref40] Jia X.; Foley A. M.; Liu C.; Vaughan B. A.; McKeown B. A.; Zhang S.; Gunnoe T. B. Styrene Production from Benzene and Ethylene Catalyzed by Palladium(II): Enhancement of Selectivity toward Styrene via Temperature-dependent Vinyl Ester Consumption. Organometallics 2019, 38 (19), 3532–3541. 10.1021/acs.organomet.9b00349.

[ref41] Bennett M. T.; Jia X.; Musgrave C. B.; Zhu W.; Goddard W. A.; Gunnoe T. B. Pd(II) and Rh(I) Catalytic Precursors for Arene Alkenylation: Comparative Evaluation of Reactivity and Mechanism Based on Experimental and Computational Studies. J. Am. Chem. Soc. 2023, 145 (28), 15507–15527. 10.1021/jacs.3c04295. 37392467

[ref42] Matsumoto T.; Periana R. A.; Taube D. J.; Yoshida H. Direct Synthesis of Styrene by Rhodium-Catalyzed Oxidative Arylation of Ethylene with Benzene. J. Catal. 2002, 206 (2), 272–280. 10.1006/jcat.2001.3471.

[ref43] Hong P.; Yamazaki H. Rhodium carbonyl-catalyzed activation of carbon-hydrogen bonds for application in organic synthesis.: V. phenylation of olefins with benzenes. J. Mol. Catal. 1984, 26 (3), 297–311. 10.1016/0304-5102(84)85102-0.

[ref44] Vaughan B. A.; Webster-Gardiner M. S.; Cundari T. R.; Gunnoe T. B. A rhodium catalyst for single-step styrene production from benzene and ethylene. Science 2015, 348 (6233), 421–424. 10.1126/science.aaa2260. 25908817

[ref45] Vaughan B. A.; Khani S. K.; Gary J. B.; Kammert J. D.; Webster-Gardiner M. S.; McKeown B. A.; Davis R. J.; Cundari T. R.; Gunnoe T. B. Mechanistic Studies of Single-Step Styrene Production Using a Rhodium(I) Catalyst. J. Am. Chem. Soc. 2017, 139 (4), 1485–1498. 10.1021/jacs.6b10658. 28106388

[ref46] Webster-Gardiner M. S.; Chen J.; Vaughan B. A.; McKeown B. A.; Schinski W.; Gunnoe T. B. Catalytic Synthesis of “Super” Linear Alkenyl Arenes Using an Easily Prepared Rh(I) Catalyst. J. Am. Chem. Soc. 2017, 139 (15), 5474–5480. 10.1021/jacs.7b01165. 28383890

[ref47] Chen J.; Nielsen R. J.; Goddard W. A.; McKeown B. A.; Dickie D. A.; Gunnoe T. B. Catalytic Synthesis of Superlinear Alkenyl Arenes Using a Rh(I) Catalyst Supported by a “Capping Arene” Ligand: Access to Aerobic Catalysis. J. Am. Chem. Soc. 2018, 140 (49), 17007–17018. 10.1021/jacs.8b07728. 30495938

[ref48] Liebov N. S.; Zhu W.; Chen J.; Webster-Gardiner M. S.; Schinski W. L.; Gunnoe T. B. Rhodium-Catalyzed Alkenylation of Toluene Using 1-Pentene: Regioselectivity To Generate Precursors for Bicyclic Compounds. Organometallics 2019, 38 (19), 3860–3870. 10.1021/acs.organomet.9b00535.

[ref49] Zhu W.; Luo Z.; Chen J.; Liu C.; Yang L.; Dickie D. A.; Liu N.; Zhang S.; Davis R. J.; Gunnoe T. B. Mechanistic Studies of Single-Step Styrene Production Catalyzed by Rh Complexes with Diimine Ligands: An Evaluation of the Role of Ligands and Induction Period. ACS Catal. 2019, 9 (8), 7457–7475. 10.1021/acscatal.9b01480.

[ref50] Jia X.; Frye L. I.; Zhu W.; Gu S.; Gunnoe T. B. Synthesis of Stilbenes by Rhodium-Catalyzed Aerobic Alkenylation of Arenes via C-H Activation. J. Am. Chem. Soc. 2020, 142 (23), 10534–10543. 10.1021/jacs.0c03935. 32453558

[ref51] Zhu W.; Gunnoe T. B. Advances in Rhodium-Catalyzed Oxidative Arene Alkenylation. Acc. Chem. Res. 2020, 53 (4), 920–936. 10.1021/acs.accounts.0c00036. 32239913

[ref52] Zhu W.; Gunnoe T. B. Rhodium-Catalyzed Arene Alkenylation Using Only Dioxygen as the Oxidant. ACS Catal. 2020, 10 (19), 11519–11531. 10.1021/acscatal.0c03439.

[ref53] Musgrave C. B.; Zhu W.; Coutard N.; Ellena J. F.; Dickie D. A.; Gunnoe T. B.; Goddard W. A. Mechanistic Studies of Styrene Production from Benzene and Ethylene Using [(η2-C2H4)2Rh(μ-OAc)]2 as Catalyst Precursor: Identification of a Bis-RhI Mono-CuII Complex As the Catalyst. ACS Catal. 2021, 11 (9), 5688–5702. 10.1021/acscatal.1c01203.

[ref54] Bennett M. T.; Reid C. W.; Musgrave C. B.; Goddard W. A.; Gunnoe T. B. Rhodium-Catalyzed Alkenylation of Arenes with Multi-Substituted Olefins: Comparison of Selectivity and Reaction Rate as a Function of Olefin Identity. Organometallics 2023, 42 (10), 908–920. 10.1021/acs.organomet.3c00073.

[ref55] Leal B. C.; Aydos G. L. P.; Netz P. A.; Dupont J. Ru-Catalyzed Estragole Isomerization under Homogeneous and Ionic Liquid Biphasic Conditions. ACS Omega 2017, 2 (3), 1146–1155. 10.1021/acsomega.7b00078. 28393133 PMC5377274

[ref56] Keith J. A.; Henry P. M. The Mechanism of the Wacker Reaction: A Tale of Two Hydroxypalladations. Angew. Chem., Int. Ed. 2009, 48 (48), 9038–9049. 10.1002/anie.200902194. 19834921

[ref57] Hoechst Reveals Wacker Process Details. Chem. Eng. News Archive 1961, 39 ( (16), ), 52–55. 10.1021/cen-v039n016.p052.

[ref58] Eckert M.; Fleischmann G.; Jira R.; Bolt H. M.; Golka K. Acetaldehyde. In Ullmann’s Encyclopedia of Industrial Chemistry; Wiley-VCH Verlag, 2012; Vol. 1, pp 1–17.

[ref59] Werner H.; Poelsma S.; Schneider M. E.; Windmüller B.; Barth D. Synthesis and Reactivity of Bis(ethene) Rhodium(I) and Iridium(I) Carboxylato Complexes. Chem. Ber. 1996, 129 (6), 647–652. 10.1002/cber.19961290609.

[ref60] Xie L.-H.; Suh M. P. Flexible Metal-Organic Framework with Hydrophobic Pores. Chem.—Eur. J. 2011, 17 (49), 13653–13656. 10.1002/chem.201103078. 22052640

