# Supplementary material for: Rhodium-Catalyzed Oxidative Alkenylation of Anisole: Control of Regioselectivity
Source: Organometallics. 2024 Jun 13;43(12):1362–76. doi: 10.1021/acs.organomet.4c00155 (PMC11200324; doi:10.1021/acs.organomet.4c00155)
Supplement: Supplementary file 1 — om4c00155_si_001.pdf [file om4c00155_si_001.pdf]

Supporting Information for

Rhodium-catalyzed Oxidative Alkenylation of Anisole: Control of Regioselectivity

Christopher W. Reid† and T. Brent Gunnoe†*

†Department of Chemistry, University of Virginia, Charlottesville, Virginia 22904, United States

*Correspondence to: tbg7h@virginia.edu

Table of Contents:

Photograph of Reactor and Reaction Setup……………………………………………………………S1

Representative GC-FID Chromatogram for Rh Catalyzed Oxidative Anisole Alkenylation………….....….S2

GC-FID Calibration Curves for 2-methoxystyrene, 3-methoxystyrene and 4-methoxystyrene……………………………………………………………………………..……………..S3

Representative GC-FID Chromatogram for Rh Catalyzed Oxidative Anisole Alkenylation with Propylene as Olefin……………………………………………………………………………..….……………………S4

GC-FID Calibration Curves for *trans*-anethole……………………………………………………………S5

Representative GC-FID Chromatogram for Rh Catalyzed Oxidative Anisole Alkenylation with Propylene as Olefin After Pd/C Hydrogenation………………………………………………………………………….S6

GC-FID Chromatograms Used for Hydrogenated Propenylanisoles Analysis……………..………………..S7

Optimization of Reaction Conditions for Anisole Alkenylation using Ethylene as the Olefin………………S8

$Cu(OPiv)_2$ Concentration Optimization for Oxidative Anisole Alkenylation…………………………………S9

Temperature and HOPiv Optimization for Oxidative Anisole Alkenylation………………………………….S10

Acid Identity Study for Oxidative Arene Alkenylation……………………………………………………….S11

Product Consumption by HOPiv Study for Oxidative Arene Alkenylation…………………………………..S12

Product Inhibition Study for Anisole Alkenylation…………………………………………………………...S13

Photograph of Reaction Progress……………………………………………………………………………S14

Control Experiment for 4-allylanisole isomerization using $Ru(Cl)_2(PPh_3)_3$………………………………….S15

Representative GC-FID Chromatogram for Rh Catalyzed Oxidative Anisole Alkenylation with Propylene as Olefin and then Isomerization using $Ru(Cl)_2(PPh_3)_3$………………………………………………………S16

Oxidative Arene Alkenylation with 50 psig Propylene and 1 atm O_2………………………………………..S17

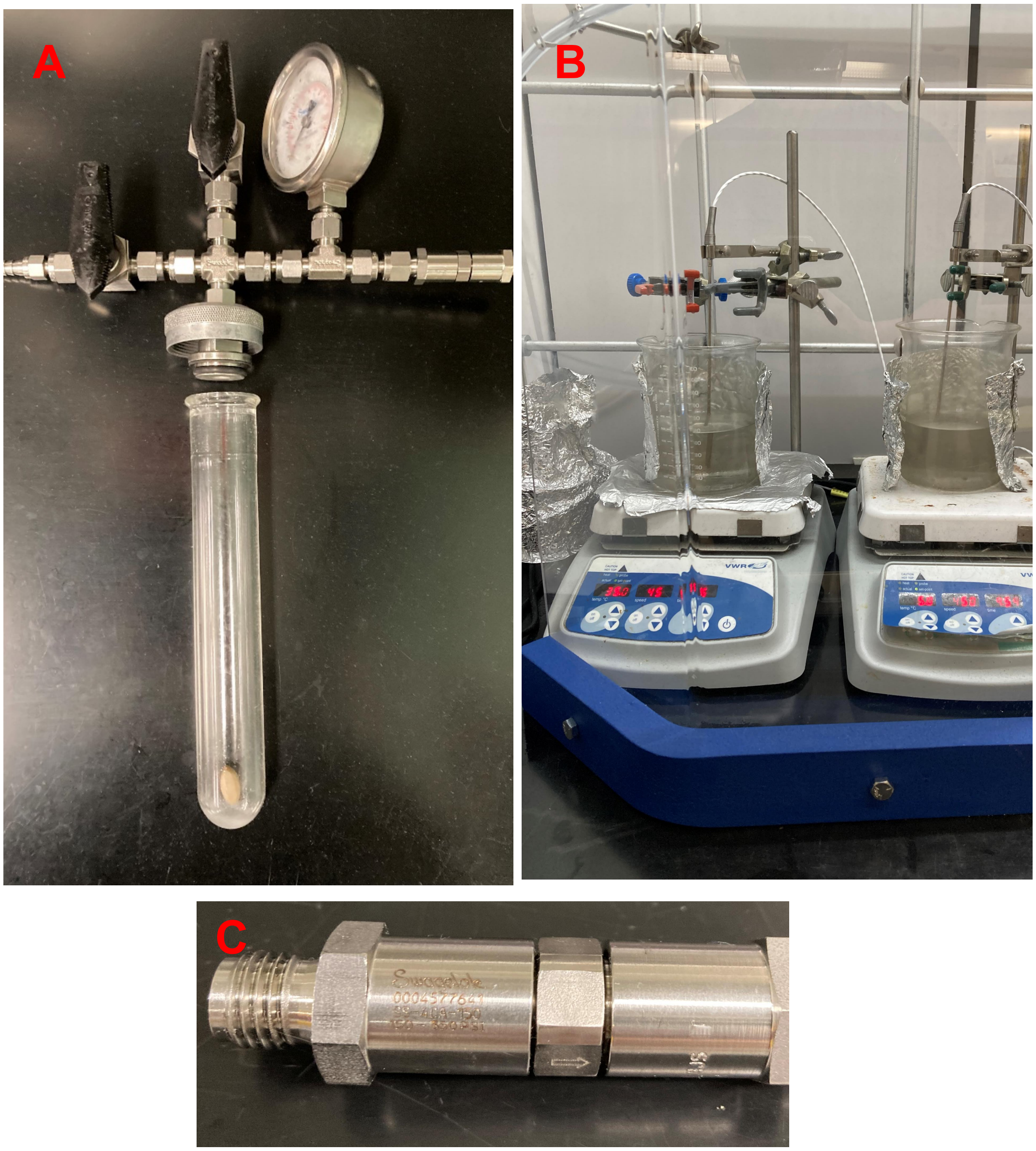

Figure S1. Photographs of: A) 3 oz Fisher-Porter reactor with reactor top fitted with adjustable pressure poppet check valve and pressure gauge , B) two oil baths behind a blast shield, and C) close-up of adjustable pressure poppet check valve.

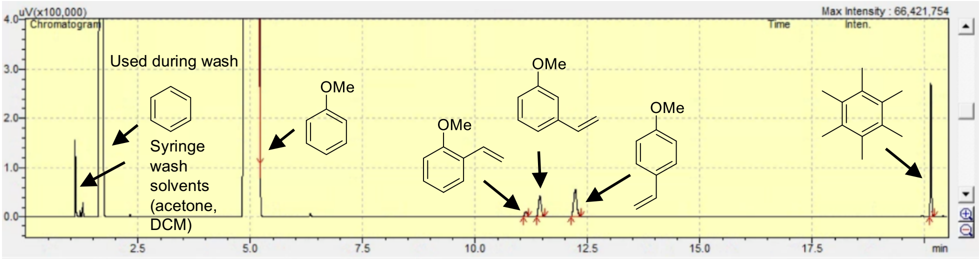

Figure S2. Representative GC-FID chromatogram for Rh catalyzed oxidative anisole alkenylation with ethylene as olefin.

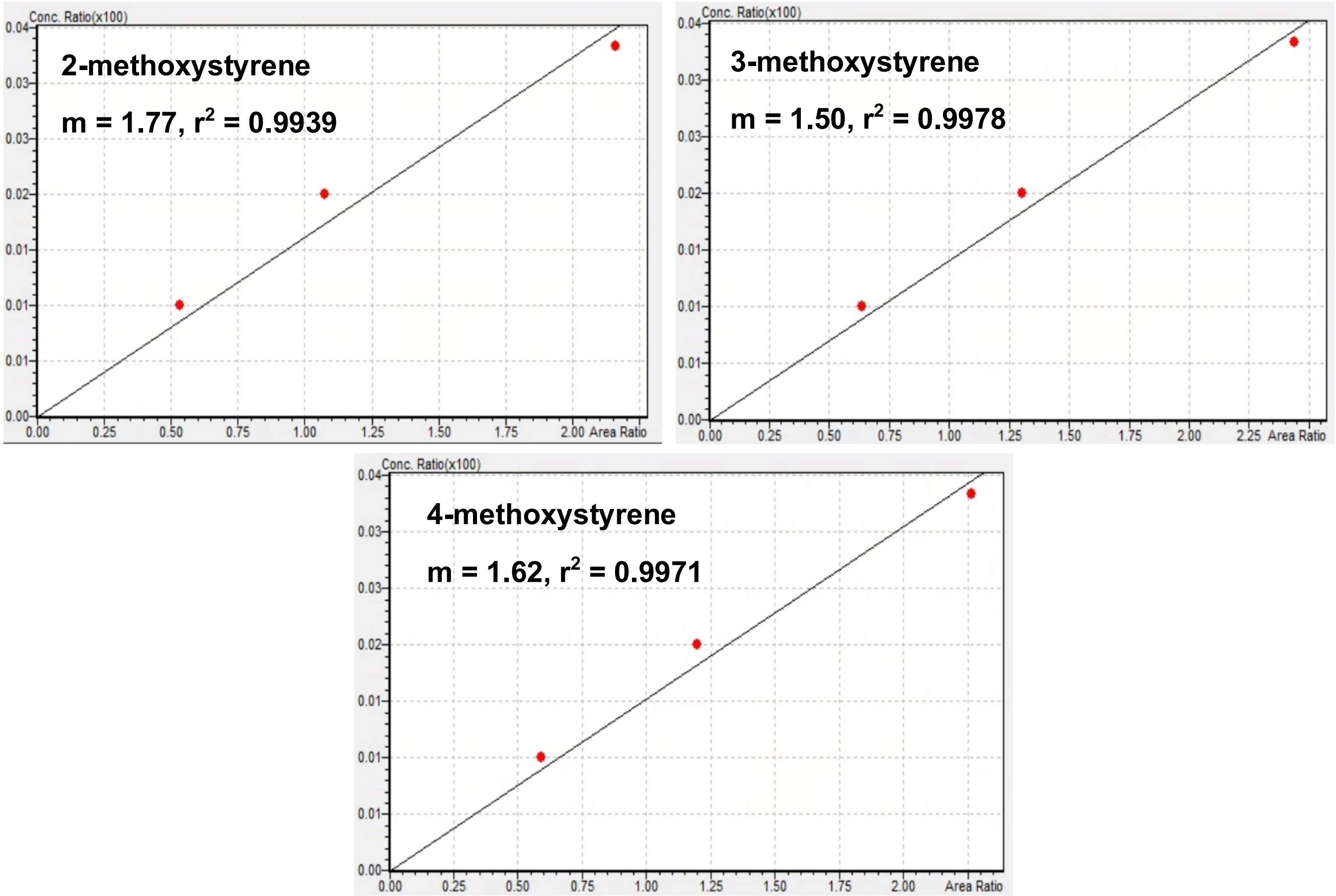

Figure S3. GC-FID calibration curves for 2-methoxystyrene, 3-methoxystyrene and 4-methoxystyrene.

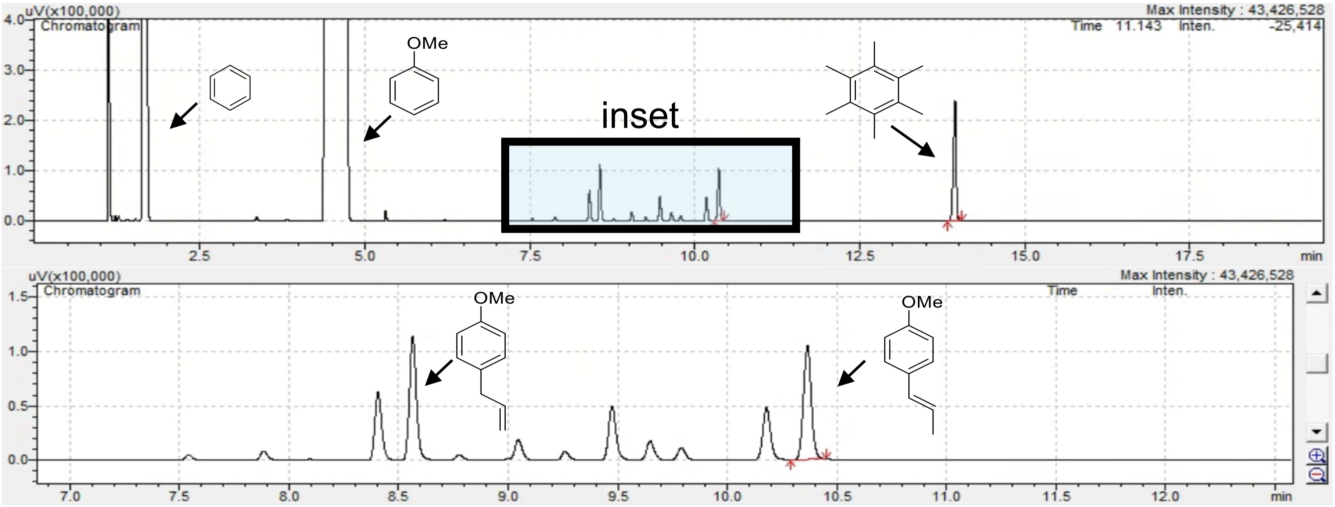

Figure S4. Representative GC-FID chromatogram for Rh catalyzed oxidative anisole alkenylation with propylene as olefin.

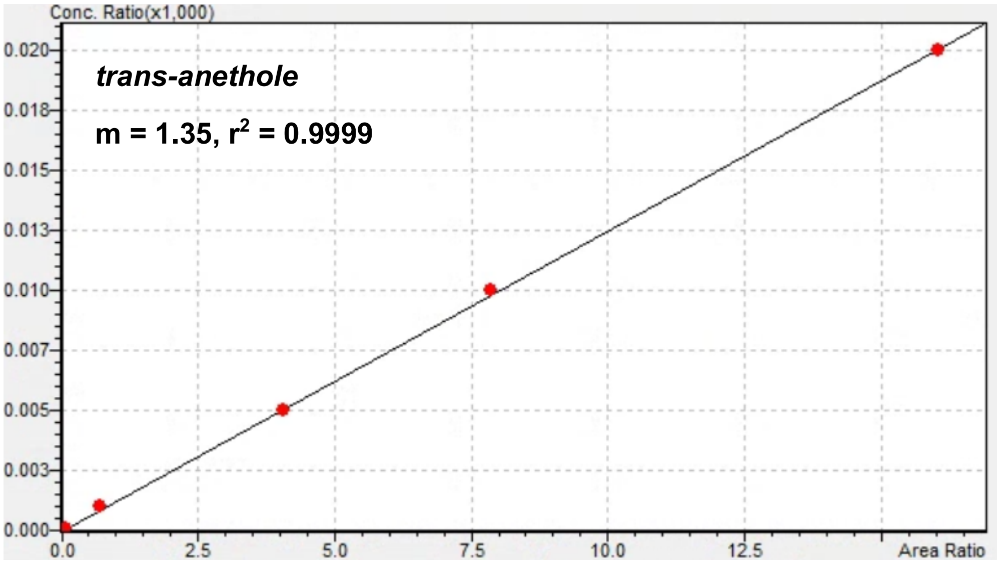

Figure S5. GC-FID calibration curves for *trans*-anethole.

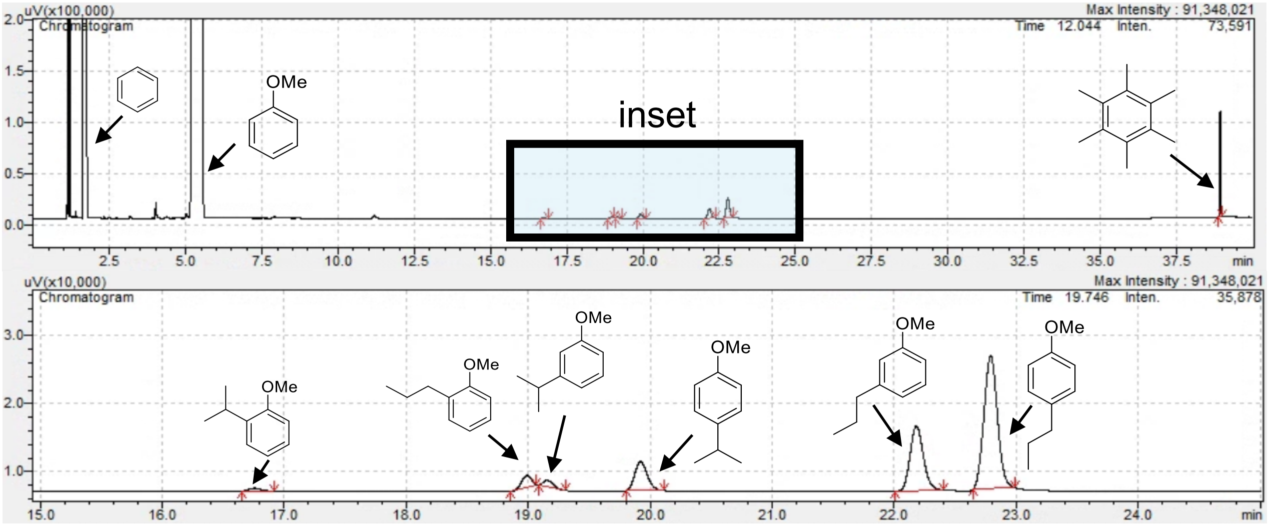

Figure S6. Representative GC-FID chromatogram for Rh catalyzed oxidative anisole alkenylation with propylene as olefin after Pd/C hydrogenation.

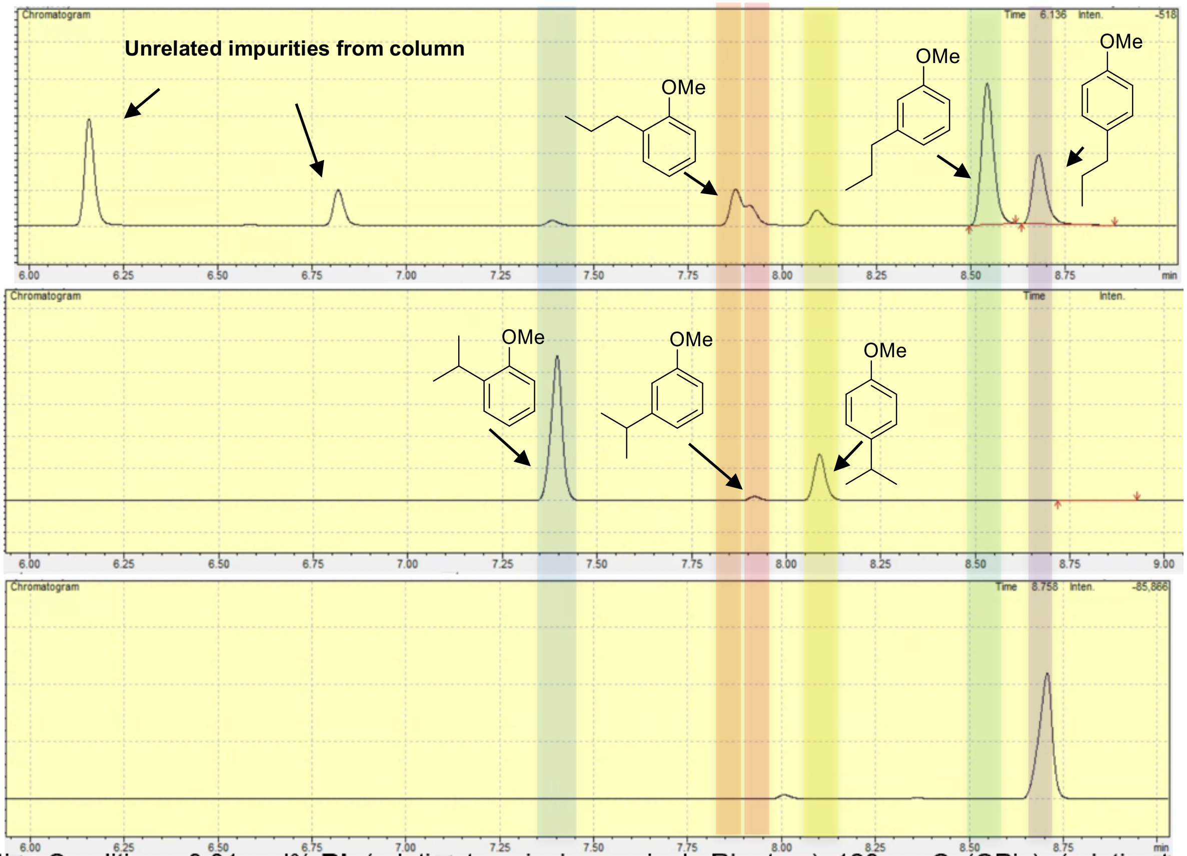

Figure S7. GC-FID chromatograms used for hydrogenated propenylanisoles analysis.

Top chromatogram in Figure S6 is a representative chromatogram of Rh catalyzed oxidative anisole alkenylation using propylene as the olefin after a Pd/C hydrogenation step. Middle chromatogram is a GC-FID from a Friedel-Crafts alkylation reaction with anisole and propylene (reaction conditions: 7.5 mL anisole, 10 mol% $AlCl_3$ relative to anisole, 50 psig propylene, 100 ºC, 30 mins). Bottom chromatogram is a GC-FID chromatogram from the hydrogenation of *trans*-anethole (Reaction conditions: 100 mg 5wt% Pd/C, 1 mL *trans*-anethole, 6.5 mL ethanol, 50 psig H_2, 50 ºC, 18 hours).

We are interested in identifying the 6 colored peaks in the top chromatogram. The 6 products are 2-, 3-, 4-isopropylanisole (branched products) and 1-methoxy-2-propylbenzene, 1-methoxy-3-propylbenzene, and 1-methoxy-4-propylbenzene (linear products). The Friedel-Crafts alkylation reaction with anisole and propylene yields 3 products, 2-isopropylanisole (blue), 3-isopropylanisole (red), and 4-isopropylanisole (yellow). The *trans*-anethole hydrogenation will yield 1-methoxy-4-propylbenzene (purple) as the sole product. By process of elimination, we assign the green highlighted peak to 1-methoxy-3-propylbenzene and the orange highlighted peak to 1-methoxy-2-propylbenzene.

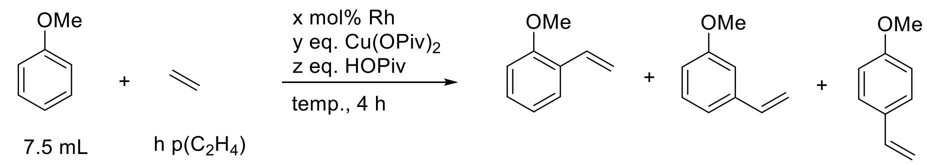

Table S8. Optimization of reaction conditions for anisole alkenylation using ethylene as the olefin.

Entry	[Rh] (mol%)	C_2H_4 (psig)	$Cu(OPiv)_2$ (eq.)	HOPiv (eq.)	Temp (ºC)	*ortho* TOs	*meta* TOs	*para* TOs	*o:m:p*	total TOs
**1**	0.01	50	120	240	150	4.2(0.2)	17.3(0.5)	9.5(0.4)	0.4:1.8:1	31(1)
**2**	0.01	50	120	240	135	2.9(0.2)	12.8(0.9)	6.2(0.3)	0.5:2.1:1	22(1.4)
**3**	0.01	50	120	240	165	5.73(0.4)	21.5(1)	14.4(1)	0.4:1.5:1	42(2)
**4**	0.01	50	120	0	150	3.76(0.2)	13.5(1)	26.1(1)	0.1:0.5:1	43(3)
**5**	0.01	50	120	600	150	3.1(0.1)	11.3(2)	3.9(0.6)	0.8:2.9:1	18(4)
**6**	0.01	50	120	1200	150	2.3(0.04)	7.6(0.1)	2.0(0.03)	1.1:3.4:1	12(0.1)
**7**	0.005	50	600	1200	150	6.1(0.5)	23.9(3)	14.0(2)	0.4:1.7:1	44(6)
**8**	0.001	50	1200	2400	150	18.3(4)	64.8(18)	41.1(15)	0.4:1.6:1	124(37)
**9**	0.001	50	1200	0	150	25.5(3)	74.8(8)	182(17)	0.1:0.4:1	283(28)
**10**	0.01	30	120	240	150	2.4(0.4)	11.2(2)	4.7(0.9)	0.6:2.4:1	19(3)
**11**	0.01	70	120	240	150	4.3(0.2)	17.5(1.4)	12.8(1)	0.3:1.4:1	35(2.5)
**12**	0.01	90	120	240	150	3.4(0.2)	14(0.3)	11.3(0.9)	0.3:1.2:1	28(1.4)
**13**	0.01	200	120	240	150	3.6(0.2)	12.8(0.6)	17.7(0.8)	0.2:0.7:1	34(1.4)

Reaction conditions: x mol% $[(\eta^2\text{-}C_2H_4)_2Rh(\mu\text{-}OAc)]_2$, 7.5 mL anisole, h psig ethylene, y eq. $Cu(OPiv)_2$, z eq. HOPiv, 150 ºC, 4 h. Catalyst loading is relative to anisole per single Rh atom. $Cu(OPiv)_2$ and HOPiv loading relative to amount of Rh catalyst. Hexamethylbenzene (HMB) used as internal standard. Standard deviations for a minimum of three independent reactions are given in parenthesis.

Representative procedure for optimization study of oxidative anisole alkenylation. A 10 mL stock solution of $[(\eta^2\text{-}C_2H_4)_2Rh(\mu\text{-}OAc)]_2$ (15.1 mg, 6.9 μmol, 1 eq. per Rh atom or other appropriate amount) was prepared in anisole. To oven-dried 3-oz Fisher-Porter reactors with adjustable pressure poppet check valves for pressure safety release,, 1 mL of stock solution, $Cu(OPiv)_2$ (120 eq. per Rh atom, 220 mg, 828 μmol or other appropriate amount), HOPiv (240 eq. per Rh atom, 169 mg, 1.65 mmol or other appropriate amount) HMB (22.4 mg, 138 μmol, 2 eq. per Rh atom) and 6.5 mL of anisole were added. The Fisher-Porter reactors were sealed and taken out of the glovebox. Ethylene (50 psig) was added to each reactor using a high-pressure gas manifold. The reaction solutions were stirred and heated at 150 °C using a silicone oil bath hot plate for 4 hours. A polycarbonate blast shield (4.7mm thickness, 30'' height) was placed in front of the stirring reactors during heating. After cooling to room temperature, an aliquot of this solution was diluted with benzene and washed with a saturated solution of $NaHCO_3$ prior to GC-FID analysis.

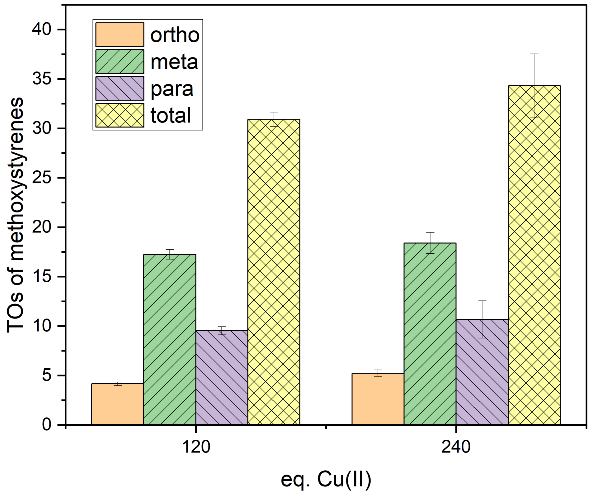

Figure S8. $Cu(OPiv)_2$ concentration optimization for oxidative anisole alkenylation. Reaction conditions: 0.01 mol% $[(\eta^2\text{-}C_2H_4)_2Rh(\mu\text{-}OAc)]_2$, 7.5 mL anisole, 50 psig ethylene, x eq. $Cu(OPiv)_2$, 240 eq. HOPiv, 150 ºC, 4 h. Catalyst loading is relative to anisole per single Rh atom. $Cu(OPiv)_2$ and HOPiv loading relative to amount of Rh catalyst. HMB used as internal standard. Error bars represent the standard deviation for a minimum of three independent reactions.

Representative procedure for $Cu(OPiv)_2$ optimization study of oxidative anisole alkenylation. A 10 mL stock solution of $[(\eta^2\text{-}C_2H_4)_2Rh(\mu\text{-}OAc)]_2$ (15.1 mg, 6.9 μmol, 1 eq. per Rh atom) was prepared in anisole. To oven-dried 3-oz Fisher-Porter reactors with adjustable pressure poppet check valves for pressure safety release, 1 mL of stock solution, $Cu(OPiv)_2$ (120 eq. or 240 eq. per Rh atom), HMB (22.4 mg, 138 μmol, 2 eq. per Rh atom) and 6.5 mL of anisole were added. The Fisher-Porter reactors were sealed and taken out of the glovebox. Ethylene (50 psig) was added to each reactor using a high-pressure gas manifold. The reaction solutions were stirred and heated at 150 °C using a silicone oil bath hot plate for 4 hours. A polycarbonate blast shield (4.7mm thickness, 30'' height) was placed in front of the stirring reactors during heating. After cooling to room temperature, an aliquot of this solution was diluted with benzene and washed with a saturated solution of $NaHCO_3$ prior to GC-FID analysis.

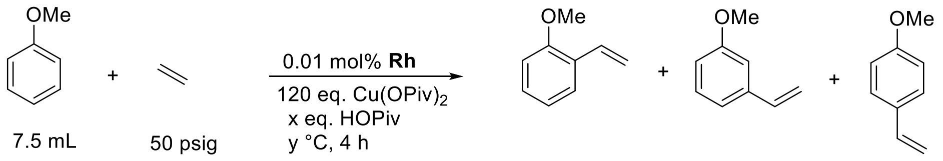

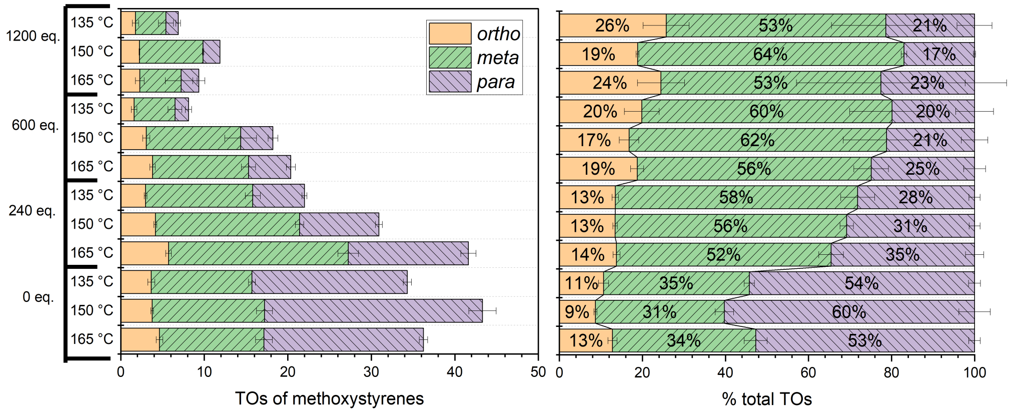

Figure S9. TOs of methoxystyrenes from oxidative anisole alkenylation using $[(\eta^2\text{-}C_2H_4)_2Rh(\mu\text{-}OAc)]_2$ as catalyst precursor (left) and % total TOs of the *ortho*, *meta*, and *para* regioisomers (right). Reaction conditions for oxidative anisole alkenylation: 0.01 mol% $[(\eta^2\text{-}C_2H_4)_2Rh(\mu\text{-}OAc)]_2$, 7.5 mL anisole, 50 psig ethylene, x eq. HOPiv, 120 eq. $Cu(OPiv)_2$, y ºC, 4 h. Catalyst loading is relative to anisole per single Rh atom. $Cu(OPiv)_2$ and HOPiv loading relative to single Rh atom. HMB used as internal standard. Error bars represent the standard deviation for a minimum of three independent reactions.

Representative procedure for temperature and HOPiv optimization studies for oxidative anisole alkenylation. A 10 mL stock solution of $[(\eta^2\text{-}C_2H_4)_2Rh(\mu\text{-}OAc)]_2$ (15.1 mg, 6.9 μmol, 1 eq. per Rh atom) was prepared in anisole. To oven-dried 3-oz Fisher-Porter reactors with adjustable pressure poppet check valves for pressure safety release, 1 mL of stock solution, $Cu(OPiv)_2$ (220 mg, 828 μmol, 120 eq. per Rh atom), HOPiv (169 mg, 1.65 mmol, 240 eq. per Rh atom or other appropriate amount), HMB (2.24 mg, 13.8 μmol, 2 eq. per Rh atom) and 6.5 mL of anisole were added. The Fisher-Porter reactors were sealed and taken out of the glovebox. Ethylene (50 psig) was added to each reactor using a high-pressure gas manifold. The reaction solutions were stirred and heated at 150 °C or other appropriate temperature using a silicone oil bath hot plate for 4 hours. A polycarbonate blast shield (4.7mm thickness, 30’’ height) was placed in front of the stirring reactors during heating. After cooling to room temperature, an aliquot of this solution was diluted with benzene and washed with a saturated solution of $NaHCO_3$ prior to GC-FID analysis.

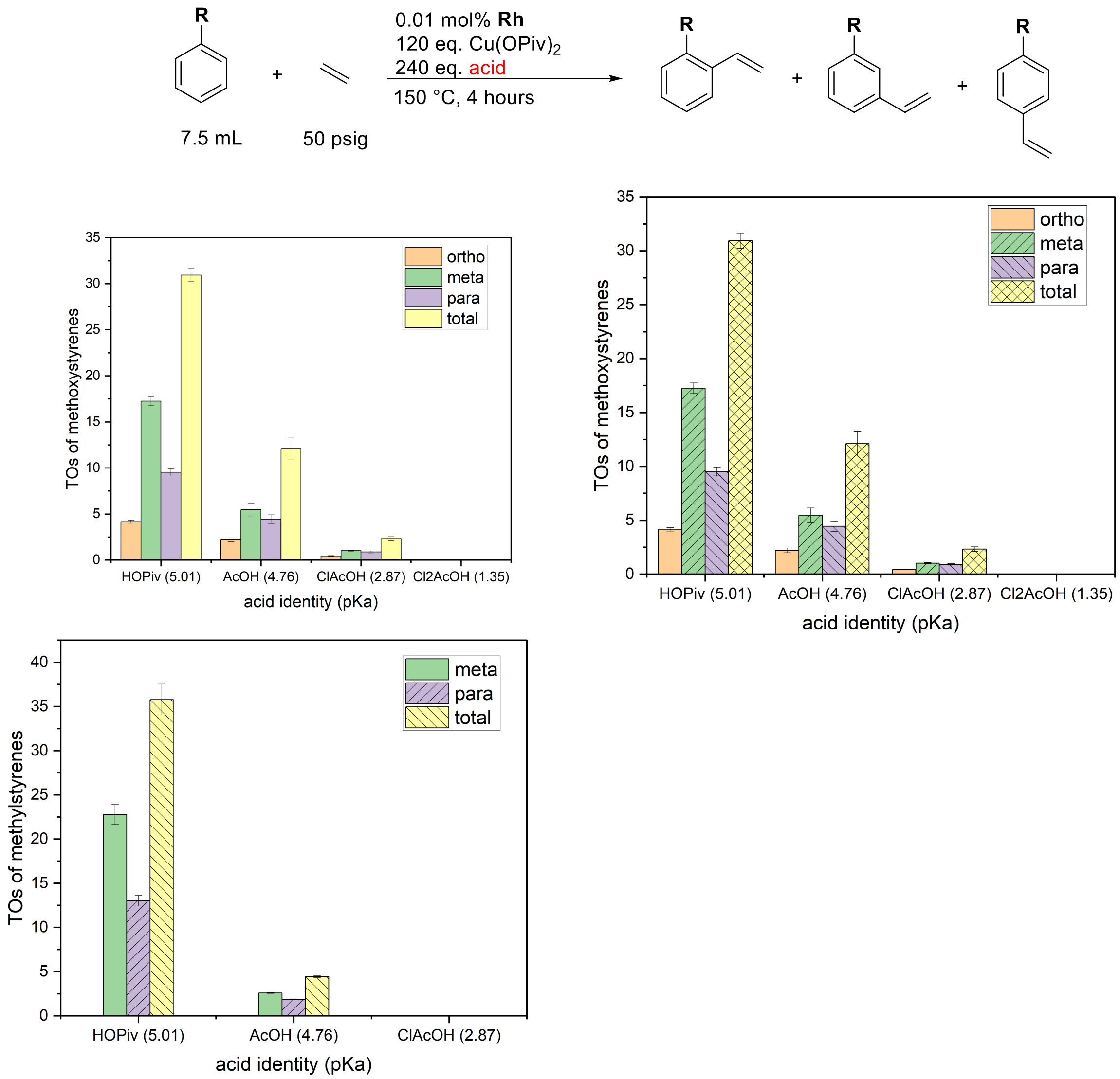

Figure S10. TOs of substituted styrenes as a function of acid identity using anisole (left) and toluene (right) as the arene and ethylene as the olefin. Reaction conditions: 0.01 mol% $[(\eta^2\text{-}C_2H_4)_2Rh(\mu\text{-}OAc)]_2$, 7.5 mL arene, 50 psig ethylene, 120 eq. Cu(**II**), 240 eq. of acid, 150 ºC, 4 h. Catalyst loading is relative to anisole per single Rh atom. $Cu(OPiv)_2$ and acid loading relative to single Rh atom. HMB used as internal standard. Error bars represent the standard deviation for a minimum of three independent reactions.

Representative procedure for acid identity study of oxidative anisole/toluene alkenylation. A 10 mL stock solution of $[(\eta^2\text{-}C_2H_4)_2Rh(\mu\text{-}OAc)]_2$ (15.1 mg, 6.9 µmol, 1 eq. per Rh atom) was prepared in either anisole or toluene. To oven-dried 3-oz Fisher-Porter reactors with adjustable pressure poppet check valves for pressure safety release, 1 mL of stock solution, $Cu(OPiv)_2$ (220 mg, 828 µmol, 120 eq. per Rh atom), HMB (22.4 mg, 138 µmol, 2 eq. per Rh atom) and 6.5 mL of anisole (or toluene) were added. The Fisher-Porter reactors were sealed and taken out of the glovebox. The appropriate amount of acetic acid (99.3 mg, 1.65 mmol, 240 eq. per Rh atom) was added to the reactor under a dinitrogen flow on a Schlenk line. Ethylene (50 psig) was added to each reactor using a high-pressure gas manifold. The reaction solutions were stirred and heated at 150 °C using a silicone oil bath hot plate for 4 hours. A polycarbonate blast shield (4.7mm thickness, 30'' height) was placed in front of the stirring reactors during heating. After cooling to room temperature, an aliquot of this solution was diluted with benzene and washed with a saturated solution of $NaHCO_3$ prior to GC-FID analysis.

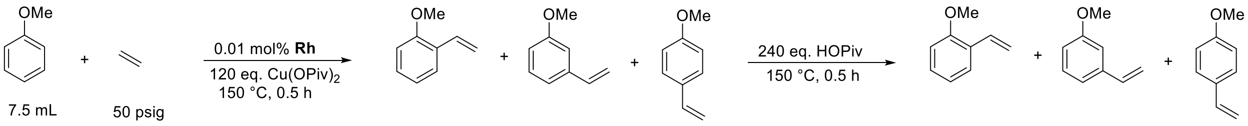

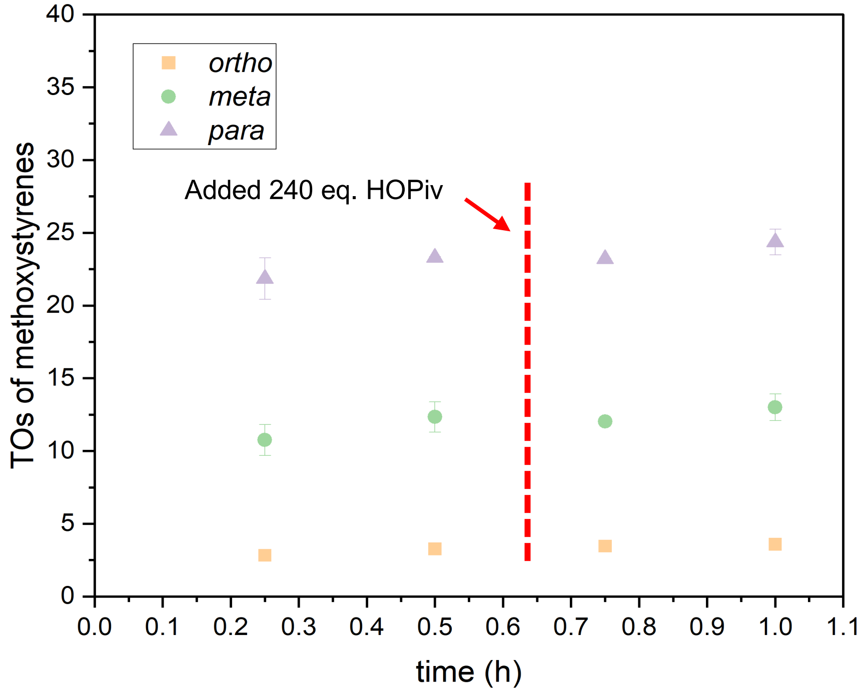

Figure S11. TOs versus time plot for oxidative anisole alkenylation using $[(\eta^2\text{-}C_2H_4)_2Rh(\mu\text{-}OAc)]_2$ as a catalyst precursor testing for product consumption. Reaction conditions: 0.01 mol% $[(\eta^2\text{-}C_2H_4)_2Rh(\mu\text{-}OAc)]_2$, 7.5 mL anisole, 50 psig ethylene, 120 eq. $Cu(OPiv)_2$, 150 ºC, 0.5 h. Then add 240 eq. HOPiv and continue reaction for another 0.5 h. Catalyst loading is relative to anisole per single Rh atom. $Cu(OPiv)_2$ and additive loading relative to single Rh atom. HMB used as internal standard. Error bars represent the standard deviation for a minimum of three independent reactions.

Representative procedure for product consumption study of oxidative anisole alkenylation. A 10 mL stock solution of $[(\eta^2\text{-}C_2H_4)_2Rh(\mu\text{-}OAc)]_2$ (15.1 mg, 6.9 µmol, 1 eq. per Rh atom) was prepared in anisole. To oven-dried 3-oz Fisher-Porter reactors with adjustable pressure poppet check valves for pressure safety release, 1 mL of stock solution, $Cu(OPiv)_2$ (220 mg, 828 µmol, 120 eq. per Rh atom), HMB (22.4 mg, 138 µmol, 2 eq. per Rh atom) and 6.5 mL of anisole were added. The Fisher-Porter reactors were sealed and taken out of the glovebox. Ethylene (50 psig) was added to each reactor using a high-pressure gas manifold. The reaction solutions were stirred and heated at 150 °C using a silicone oil bath hot plate. A polycarbonate blast shield (4.7mm thickness, 30'' height) was placed in front of the stirring reactors during heating. Aliquots of this solution were taken at 0.25 and 0.5 hours, diluted with benzene, washed with $NaHCO_3$, prior to GC-FID analysis. The appropriate amount of HOPiv (169 mg, 1.65 mmol, 240 eq. per Rh atom) was added to the reactor under a dinitrogen flow on a Schlenk line. Ethylene was again added to each reactor using a high-pressure gas manifold. Reactors were stirred and heated at 150 °C using a silicone oil bath hot plate. Aliquots of this solution were taken at 0.75 and 1 hours, diluted with benzene and washed with $NaHCO_3$ prior to GC-FID analysis.

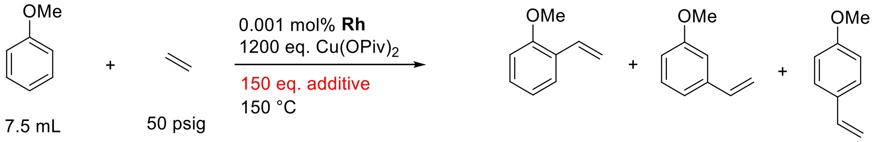

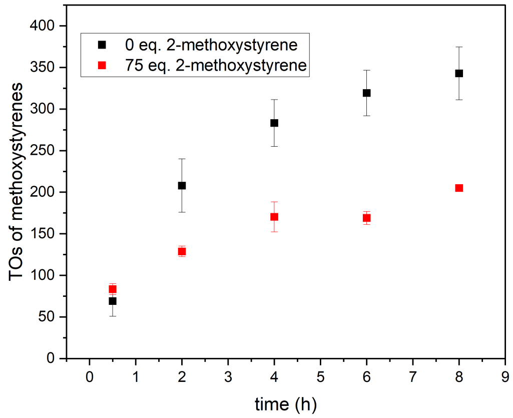

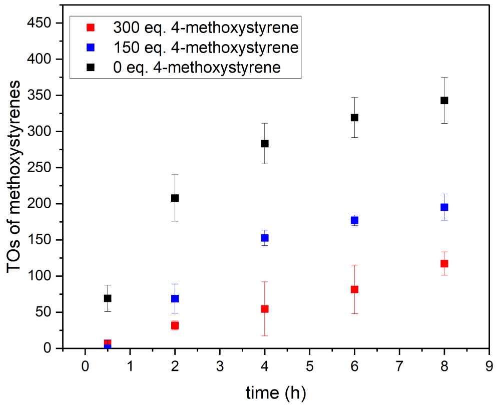

Figure S12. TOs versus time plot for oxidative anisole alkenylation using $[(\eta^2\text{-}C_2H_4)_2Rh(\mu\text{-}OAc)]_2$ as a catalyst precursor testing for product inhibition. Reaction conditions: 0.001 mol% $[(\eta^2\text{-}C_2H_4)_2Rh(\mu\text{-}OAc)]_2$, 7.5 mL anisole, 50 psig ethylene, 1200 eq. $Cu(OPiv)_2$. x eq. of additive, 150 ºC, 4 h. Catalyst loading is relative to anisole per single Rh atom. $Cu(OPiv)_2$ and additive loading relative to single Rh atom. HMB used as internal standard. TOs adjusted to account for methoxystyrene being used as an additive in the reaction. Error bars represent the standard deviation for a minimum of three independent reactions.

Representative procedure for longevity study of oxidative anisole alkenylation. A 10 mL stock solution of $[(\eta^2\text{-}C_2H_4)_2Rh(\mu\text{-}OAc)]_2$ (1.51 mg, .69 μmol, 1 eq. per Rh atom) was prepared in anisole. To oven-dried 3-oz Fisher-Porter reactors with adjustable pressure poppet check valves for pressure safety release, 1 mL of stock solution, $Cu(OPiv)_2$ (220 mg, 828 μmol, 1200 eq. per Rh atom), HMB (22.4 mg, 138 μmol, 20 eq. per Rh atom) and 6.5 mL of anisole were added. The Fisher-Porter reactors were sealed and taken out of the glovebox. The appropriate amount of 2- or 4-methoxystyrene was added to the reactor under a dinitrogen flow on a Schlenk line. Ethylene (50 psig) was added to each reactor using a high-pressure gas manifold. The reaction solutions were stirred and heated at 150 °C using a silicone oil bath hot plate. A polycarbonate blast shield (4.7mm thickness, 30'' height) was placed in front of the stirring reactors during heating. After 0.5 hours and subsequently every 2 hours, the reactors were allowed to cool

and under dinitrogen flow an aliquot of the reaction was taken, diluted with benzene and washed with a saturated solution of $NaHCO_3$ prior to GC-FID analysis. After taking the aliquot, the reactors were charged with ethylene (50 psig) and heated at 150 °C until the next time point.

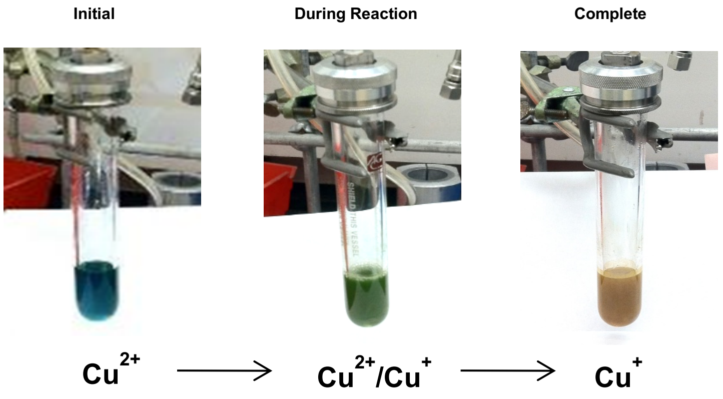

Figure S13. Photograph of reaction color as the reaction progresses. The conversion of Cu(II) to Cu(I) can qualitatively be monitored by looking at the color. Solutions comprised of all or mostly Cu^{II} are blue. Solutions comprised of all or mostly Cu^{I} are bronze/brown. Solutions that are a mixture of Cu^{II}/Cu^{I} are green.

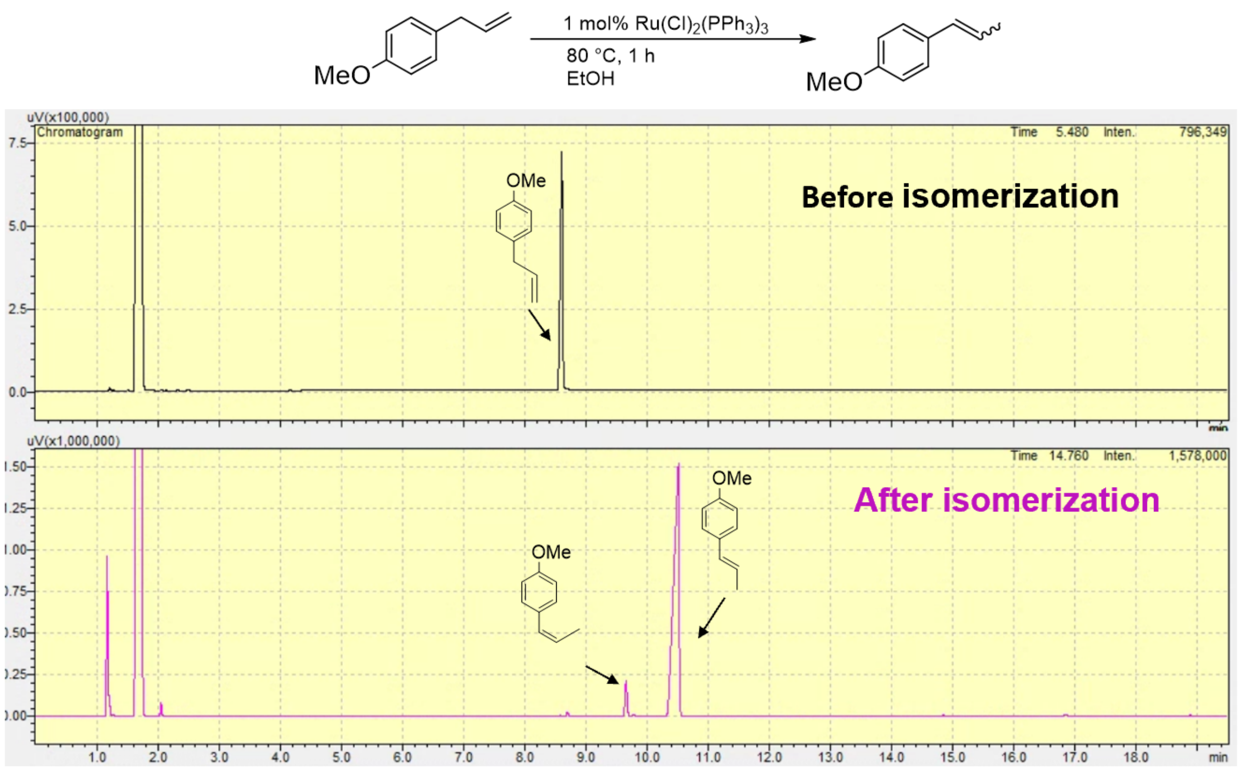

Figure S14. GC-FID chromatogram for the control experiment testing the isomerization of 4-allylanisole to *cis/trans*-anethole using 1 mol% $Ru(Cl)_2(PPh_3)_3$ as the catalyst precursor.

Representative procedure for isomerization of 4-allylanisole. To oven-dried 3-oz Fisher-Porter reactors with adjustable pressure poppet check valves for pressure safety release, 1 mL of 4-allylanisole (6.5 mmol) was added to 7.5 mL ethanol and 61 mg $Ru(Cl)_2(PPh_3)_3$ (1 mol% relative to 4-allylanisole). The Fisher-Porter reactors were sealed and taken out of the glovebox. The reaction solutions were stirred and heated at 80 °C using a silicone oil bath hot plate. A polycarbonate blast shield (4.7mm thickness, 30'' height) was placed in front of the stirring reactors during heating. After 1 hour, the reactors were allowed to cool and under dinitrogen flow an aliquot of the reaction was taken, diluted with benzene and washed with a saturated solution of $NaHCO_3$ prior to GC-FID analysis.

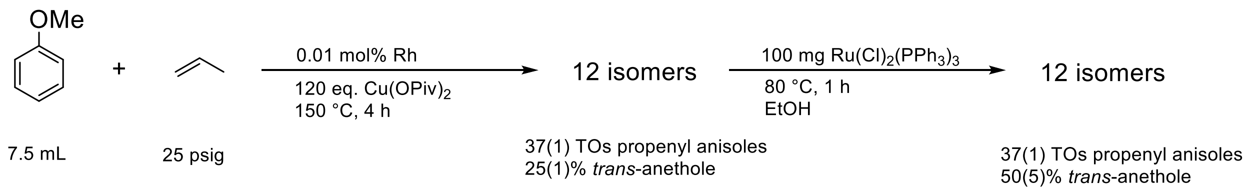

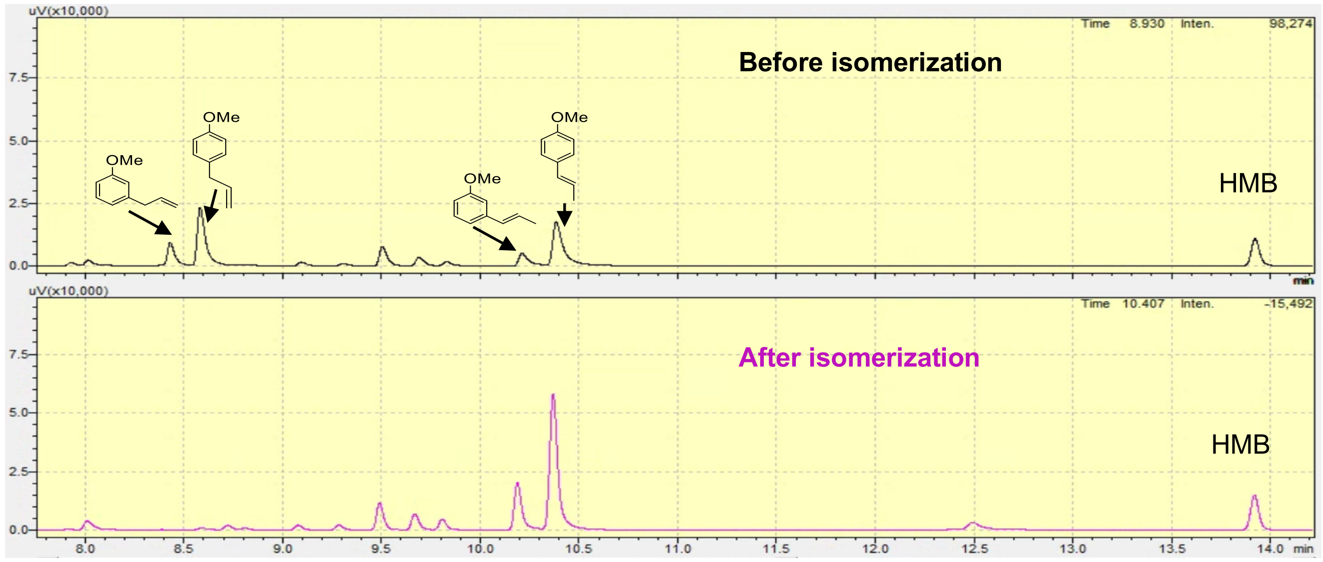

Figure S15. Representative GC-FID chromatograms for oxidative anisole alkenylation with propylene as the olefin before isomerization conditions (top) and oxidative anisole alkenylation with propylene as the olefin after isomerization conditions (bottom).

Representative procedure for oxidative anisole alkenylation using propylene as olefin and isomerization. A 10 mL stock solution of $[(\eta^2\text{-}C_2H_4)_2Rh(\mu\text{-}OAc)]_2$ (15.1 mg, 6.9 μmol, 1 equiv. per Rh atom) was prepared in anisole. To oven-dried 3-oz. Astraglass Innovations Fisher-porter reactors attached with adjustable pressure poppet check valves for pressure safety release, 1 mL of stock solution, $Cu(OPiv)_2$ (220 mg, 828 μmol, 120 equiv. per Rh atom), HOPiv (169 mg, 1.65 mmol, 240 equiv. per Rh atom), HMB (2.24 mg, 13.8 μmol, 2 equiv. per Rh atom) and 6.5 mL of anisole (total 7.5 mL reaction solution) were added. The Fisher-porter reactors were sealed and taken out of the glovebox. Propylene (25 psig) was added to each reactor using a high pressure gas manifold. Reactors were stirred and heated at 150 °C using a silicone oil bath hot plate for 4 hours. A polycarbonate blast shield (4.7mm thickness, 30" height) was placed in front of the stirring reactors during heating. After cooling to room temperature, an aliquot of this solution was diluted with benzene and washed with a saturated solution of NaHCO3 prior to GC-FID analysis. To the reaction mixture, 100 mg $Ru(Cl)_2(PPh_3)_3$ in 7.5 mL ethanol was added. Reactors were stirred and heated at 80 °C using a silicone oil bath hot plate for 1 hour. A polycarbonate blast shield (4.7mm thickness, 30" height) was placed in front of the stirring reactors during heating. After cooling to room temperature, an aliquot of this solution was diluted with benzene and washed with a saturated solution of NaHCO3 prior to GC-FID analysis.

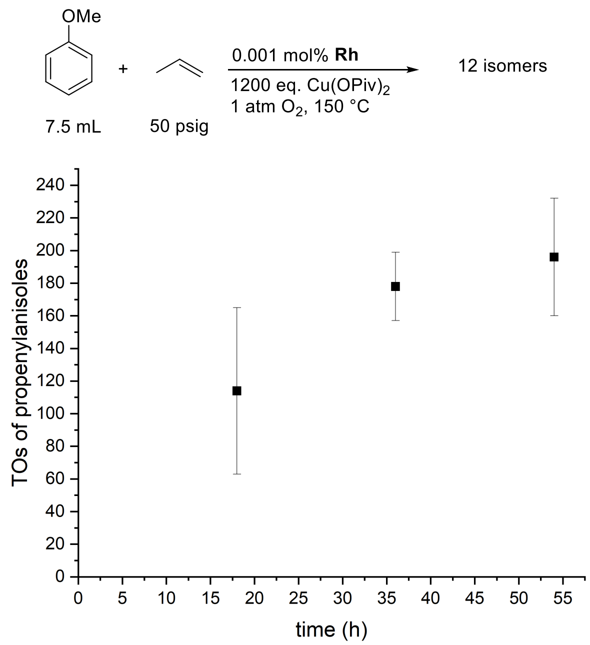

Figure S16. TOs of propenylanisoles from oxidative anisole alkenylation using $[(\eta^2\text{-}C_2H_4)_2Rh(\mu\text{-}OAc)]_2$ as catalyst precursor with 1 atm O_2 and $Cu(OPiv)_2$ as the oxidant. Reaction conditions for oxidative anisole alkenylation: 0.001 mol% $[(\eta^2\text{-}C_2H_4)_2Rh(\mu\text{-}OAc)]_2$, 7.5 mL anisole, 50 psig propylene, 1200 equiv. $Cu(OPiv)_2$. Catalyst loading is relative to anisole per single Rh atom. $Cu(OPiv)_2$ loading relative to single Rh atom. HMB used as internal standard. Error bars represent the standard deviation for a minimum of three independent reactions.

Representative procedure for oxidative anisole alkenylation using propylene as olefin with 1 atm O_2. A 10 mL stock solution of $[(\eta^2\text{-}C_2H_4)_2Rh(\mu\text{-}OAc)]_2$ (1.51 mg, .69 μmol, 1 equiv. per Rh atom) was prepared in anisole. To oven-dried 4 dram vials, 1 mL of stock solution, $Cu(OPiv)_2$ (220 mg, 828 μmol, 120 equiv. per Rh atom), HMB (2.24 mg, 13.8 μmol, 20 equiv. per Rh atom) and 6.5 mL of anisole (total 7.5 mL reaction solution) were added. The vials were inserted into a stainless steel reactor fitted with a proportional relief valve. The stainless steel reactors were hand tightened, taken out of the glovebox, and sealed with a vice. 1 atm O_2 was purged through the reactors for 5 minutes. Propylene (50 psig) was added to each reactor using a high pressure gas manifold. Reactors were stirred and heated at 150 °C using a silicone oil bath hot plate for 18 hours. A polycarbonate blast shield (4.7mm thickness, 30'' height) was placed in front of the stirring reactors during heating. After cooling to room temperature, an aliquot of this solution was diluted with benzene and washed with a saturated solution of $NaHCO_3$ prior to GC-FID analysis.
